# Spoilage Investigation of Chill Stored Meagre (*Argyrosomus regius*) Using Modern Microbiological and Analytical Techniques

**DOI:** 10.3390/foods10123109

**Published:** 2021-12-15

**Authors:** Faidra Syropoulou, Foteini F. Parlapani, Dimitrios A. Anagnostopoulos, Anastasios Stamatiou, Athanasios Mallouchos, Ioannis S. Boziaris

**Affiliations:** 1 Laboratory of Marketing and Technology of Aquatic Products and Foods, Department of Ichthyology and Aquatic Environment, School of Agricultural Sciences, University of Thessaly, Fytokou Street, 38446 Volos, Greece; faisyropou@uth.gr (F.S.); fwparlap@uth.gr (F.F.P.); anagnostopoulos.dimitriosa@gmail.com (D.A.A.); 2 Laboratory of Microbiology and Biotechnology of Foods, Department of Food Science and Human Nutrition, Agricultural University of Athens, 75 Iera Odos, 11855 Athens, Greece; anstamatiou@aua.gr; 3 Laboratory of Food Chemistry and Analysis, Department of Food Science and Human Nutrition, Agricultural University of Athens, 75 Iera Odos, 11855 Athens, Greece; amallouchos@aua.gr

**Keywords:** fish, meagre, spoilage, 16S NGS, volatile organic compounds

## Abstract

Spoilage status of whole and filleted chill-stored meagre caught in January and July was evaluated using sensory, microbiological, 16S metabarcoding and Volatile Organic Compounds (VOCs) analysis. Based on the sensory analysis, shelf-life was 15 and 12 days for the whole fish taken in January and July, respectively, while 7 days for fish fillets of both months. For the whole fish, Total Viable Counts (TVC) at the beginning of storage was 2.90 and 4.73 log cfu/g for fish caught in January and July respectively, while it was found about 3 log cfu/g in fish fillets of both months. The 16S metabarcoding analysis showed different profiles between the two seasons throughout the storage. *Pseudomonas* (47%) and *Psychrobacter* (42.5%) dominated in whole meagre of January, while *Pseudomonas* (66.6%) and *Shewanella* (10.5%) dominated in fish of July, at the end of shelf-life. Regarding the fillets, *Pseudomonas* clearly dominated at the end of shelf-life for both months. The volatile profile of meagre was predominated by alcohols and carbonyl compounds. After univariate and multivariate testing, we observed one group of compounds (trimethylamine, 3-methylbutanoic acid, 3-methyl-1-butanol) positively correlating with time of storage and another group with a declining trend (such as heptanal and octanal). Furthermore, the volatile profile seemed to be affected by the fish culturing season. Our findings provide insights into the spoilage mechanism and give information that helps stakeholders to supply meagre products of a high-quality level in national and international commerce.

## 1. Introduction

Over the last three decades (since the late 90′s), meagre *Argyrosomus regius* (Asso, 1801) constitutes one of the most interesting fish species for the Mediterranean aquaculture e.g., France, Italy, Spain, Portugal, Turkey, Greece, and Egypt [1]. The interest for meagre farming is due to its biological features e.g., fast growth of ~1 Kg per annum in captivity, good feed conversion ratio (almost like Atlantic salmon), capacity to tolerate under changing environmental conditions, less susceptibility to diseases compared to other important fish species such as sea bream, European sea bass and Senegalese sole [1,2,3], and quality characteristics, e.g., high fillet yield (42.2%) [4], low quantities of mesenteric and muscular fat than other farmed fish, good shelf-life at low storage temperatures, excellent taste, and good fatty acid profile; rich in long-chain omega-3 polyunsaturated fatty acids such as Eicosapentaenoic Acid (EPA) and Docosahexaenoic Acid (DHA) [1,5,6]. Even after cooking by boiling, meagre fillet can maintain EPA and DHA at adequate levels for the human diet, although cooking leads to lipids loss [5]. In market, meagre is mainly sold fresh (whole gutted or un-gutted stored in ice, and fillets, or slices at low temperatures). 

Fresh fish spoils faster than other foods of animal origin, due to microbial activity. Microorganisms in fish come from the environment wherein fish lives and from various sources of contamination in post-farm gate e.g., harvesting, handling, packaging, processing, and distribution [7]. Bacterial communities of fresh fish are much affected by post-harvest practices, such as handling, gutting, and filleting [8]. In storage, temperature and packaging conditions determine the predominant spoilage microbiota of fish. A small consortium of bacterial genera or species of the initial total microbiota of fish cause the deterioration of quality. These bacteria, the so-called Specific Spoilage Organisms (SSOs), present higher growth rates than the rest microbiota, under specific storage conditions, and produce metabolites responsible for off-odors and off-flavors when their population reaches the spoilage levels of 7–9 logs [9,10,11]. The production of Volatile Organic Compounds (VOCs) mainly alcohols, aldehydes, ketones, organic acids, and esters has been linked with quality deterioration of fish during storage [9]. Such information is well known for a variety of seafoods, also including the gilt-head seabream and the European sea bass [12,13,14]; the most important fish species of the Mediterranean aquaculture. In the literature, there is only one publication on the volatile profile of meagre, but it concerns the raw fish before storage [15] and not the changes during storage. 

Until last decade, the determination of seafood spoilage microbiota was routinely studied by both culture-dependent [16,17] and culture-independent techniques [18,19,20,21]. However, the advent of Next Generation Sequencing (NGS) and specifically the application of 16S rRNA amplicon-based metabarcoding analysis has revolutionized the way of seafood spoilage microbiota evaluation, providing a clear snapshot of microbial communities present, highlighting in parallel the presence of many bacterial groups in several seafood products, that escaped from conventional approaches [22,23,24]. It is crucial to point out that this method reveals both cultivable and non-cultivable microbiota directly from seafood and thus, it constitutes a rational way to enrich the current knowledge about the microbiota formation during storage and more importantly, at the end of fish shelf-life.

To our knowledge, no information is available in the literature, regarding the spoilage analysis during storage of meagre (whole and filleted), using such modern microbiological and analytical techniques. The aim of this study was (a) to record sensory, microbial population, and chemical changes, particularly VOCs determination using headspace Solid Phase Micro-Extraction—Gas Chromatography/Mass Spectrometry (SPME-GC/MS), and (b) to assess microbial communities’ composition through 16S metabarcoding analysis, in whole and filleted meagre during storage at 0 (ice) and 4 °C, respectively, to get information for the spoilage status of this kind of fish. The importance of determining the microbial and VOCs profile during storage (and more importantly at the end of shelf-life) of a food product is undeniable, while the use of modernized techniques to unveil the hitherto “unknown profile” of such kind of fish, is of great industrial and scientific interest.

## 2. Materials and Methods

### 2.1. Provision, Storage, and Sampling of Meagre

Samples from two batches of whole and filleted meagre (*Argyrosomus regius*) of approximately 2 kg and 500 g, respectively, were provided from a leading Greek aquaculture company in two different seasons January and July 2020. Fish were harvested from aquaculture farm (at day 0), transferred to the processing unit, handled as whole or filleted and finally packed (whole fish and fillets) in insulated boxes with melted ice. Then the samples were transferred to the Laboratory of Marketing and Technology of Aquatic Products and Foods (University of Thessaly, Volos) and received at day 2 after harvest, simulating a typical distribution time of the product. Whole meagre was stored in melted ice (0 °C) while fish fillets were stored in incubators operating at 4 °C to simulate the commercial storage conditions.

At each sampling point three whole fish and three fillets per batch (three fish x two batches = six whole fish or fillets) were taken per sampling month (January or July). Sampling for sensory and microbiological analysis was carried out every two days for fish fillets (Days 3, 5, 7, 9, 11), while every three days for the whole fish (Days 3, 6, 9, 12, 15, 18). Sampling for 16S metabarcoding analysis and for the determination of VOCs was carried out at four time points, from the beginning of storage until the end of fish shelf-life, especially in Day (D) 3, 6, 12 and 15 for the whole fish, while in D 3, 5, 7 and 9 for the fish fillets. The samples for 16S metabarcoding analysis and VOCs determination were stored at −20 °C until the analysis. 

### 2.2. Sensory Evaluation of Fish

The aim of the sensory evaluation was to determine the rejection time point. Five trained panelists evaluated the sensory attributes (e.g., skin appearance, and odor of flesh) of the whole and filleted meagre. The overall quality rating of whole fish was assigned on a 5 to 1 scale with 5, 4, 3 and 2 corresponding to the categories E, A, B and C, respectively, according to Multilingual Guide to EU Freshness Grades for Fishery Products [25], while score 1 was attributed to a totally spoiled sample. Average score below 3 was considered as the score for rejection. The sensory attributes of fillets were appearance (translucent, glossy, natural color, opaque, dull, discolored) and odor (marine, fresh, neutral, sour, stale, spoiled, putrid). Each sensory attribute was rated using a five-point descriptive hedonic scale (5 being the highest quality score and 1 the lowest). An average score below 3 was taken as the score for minimum acceptability. 

### 2.3. Microbiological Analysis 

Twenty-five grams (25 g) of fish tissue were taken aseptically from six fish or fillets (three from each batch) and then transferred to stomacher bags with 225 mL MRD (Maximum Recovery Diluent, 0.1% *w*/*v* peptone, 0.85% *w*/*v* NaCl) and homogenized for 2 min using a Stomacher (Bug Mixer, Interscience, London, UK). Using spread plate technique, an amount of 0.1 mL of 10-fold serial dilutions was applied on the surface of dried media in Petri dishes for enumeration of Total Viable Counts (TVC) on TSA (Tryptone Soy Agar), incubated at 25 °C for 48–72 h, and *Pseudomonas* spp. on cetrimide-fucidin-cephaloridine agar (CFC) incubated at 25 °C for 48 h. Using pour plate technique, 1 mL of 10-fold serial dilutions was used for the enumeration of H_2_S producing bacteria on Iron Agar (IA) by counting only black colonies after incubation at 25 °C for 72 h, Enterobacteriaceae on Violet Red Bile Glucose agar (VRBGA), after incubation at 37 °C for 24 h, and Lactic Acid Bacteria on De Man, Rogosa, Sharpe agar (MRS) with initial pH adjusted to 6.4 and 8, after incubation at 25 °C for 72 h. All microbiological media were supplied from LAB M (Lancashire, UK). The results were expressed as mean log cfu g^−1^ ± standard deviation (log colony forming unit per g) of six replicates. 

### 2.4. 16S Metabarcoding

#### 2.4.1. Samples Preparation and DNA Extraction 

Before DNA extraction, 25 g of pooled sample were taken from six fish or fillets (three from each batch) and transferred aseptically to stomacher bags with 225 mL sterile saline solution (0.85% *w*/*v*, 1:2 dilution) and homogenized for 4 min in a Stomacher. A volume of 225 mL of homogenized fish suspension was transferred aseptically to sterile centrifuge tubes and centrifuged at 136× *g* for 5 min at 20 °C (NF 400R bench top refrigerated centrifuge, Nuve, Turkey) to remove any fish particles. Avoiding the lipid interface, the supernatant was transferred to sterile centrifuge tubes and centrifuged at 2067× *g* for 15 min at 20 °C. Finally, the pellet was diluted in 1 mL of sterile deionized H_2_O.

For each sample, 200 μL of diluted pellet were used for bacterial DNA extraction with the NucleoSpin Tissue kit (Macherey-Nagel GmbH & Co. KG, Düren, Germany). According to the manufacturer’s instructions. DNA concentration was measured on a nanodrop Quawell UV-Vis Spectrophotometer Q5000 (Quawell Technology, Inc., San Jose, CA, USA).

#### 2.4.2. Library Preparation, Sequencing and Bioinformatic Analysis

The metagenetic analysis was applied by amplifying the V1-V3 loci of 16S rRNA gene, using the primers 27F (AGRGTTTGATCMTGGCTCAG) and 519Rmodbio (GWATTACCGCGGCKGCTG). Each sample underwent a single-step 30 cycle PCR using HotStarTaq Plus Master Mix Kit (Qiagen, Valencia, CA, USA). The conditions of PCR were as follows: 95 °C for 5 min, followed by 30 cycles of 95 °C for 30 s, 53 °C for 40 s and 72 °C for 1 min, followed by a final elongation at 72 °C for 10 min. Thereafter, all samples’ amplicons were mixed in equal concentrations, purified using SPRI beads and sequenced on a MiSeq Illumina platform according to manufacturer’s protocols.

Raw sequence data were processed using the MR DNA ribosomal and functional gene analysis pipeline (www.mrdnalab.com, accessed on 10 September 2021, MR DNA, Shallowater, TX, USA). Only the high-quality sequences (≥Q25) were depleted of primers, while short sequences (<150 bp) and sequences with ambiguous base calls were removed. They were further processed by quality filtering using a maximum expected error threshold of 1.0, followed by dereplication. The dereplicated or unique sequences were denoised, while unique sequences identified with sequencing or PCR point errors were removed, followed by chimera check, to provide denoised sequence (zOTU). Finally, zOTUs were taxonomically classified using BLASTn against a curated database derived from the National Center for Biotechnology Information (NCBI) (www.ncbi.nlm.nih.gov, accessed on 13 September 2021) and generated into relative abundances at different taxonomic levels (from phylum to genus). The estimation of alpha and beta diversity was applied as described previously [26,27,28,29,30] using Quantitative Insights Into Microbial Ecology 2 (Qiime 2) [31]. All samples were rarefied to 5000 sequences using the DADA2 algorithm. Alpha rarefaction curve was plotted with 10 sampling depths. For beta diversity, a principal coordinate analysis (PCoA) plot was generated based on the weighted UniFrac distance.

The raw sequences were deposited in the National Centre for Biotechnology Information (NCBI), under the Bioproject PRJNA749170. 

### 2.5. Determination of Volatile Compounds by Headspace SPME-GC/MS

A small portion (approximately 5 g) of fish tissue was taken from six fish or fillets (three from each batch) in triplicates and cut quickly in small cubes, snap-frozen in liquid nitrogen to quench metabolism and grinded for 10–15 s in a pre-cooled A11 analytical mill (IKA, Wilmington, NC, USA) to obtain a fine frozen powder. Aliquots (2 g) of each powdered sample were accurately weighed (±0.01 g) in a porcelain mortar containing 2 g (NH_4_)_2_SO_4_, homogenized for 20 s and transferred into 20 mL headspace glass vial. The vials were sealed with crimp caps with PTFE-lined silicone septa and equilibrated for 15 min at 40 °C in a water bath. The volatiles were extracted by exposing the SPME fiber (DVB/CAR/PDMS, length 2 cm, Sigma Aldrich, Taufkirchen, Germany) for 30 min under the same conditions. The volatiles were desorbed in the injection port of a GCMS-QP2010 Ultra (Shimadzu Inc., Tokyo, Japan) equipped with a SPME liner (0.7 mm i.d.) at 240 °C in splitless mode for 5 min. Afterwards, the fiber was retracted and conditioned for 5 min at 250 °C in the injection port of another GC to remove any volatile residues. The separation of the compounds was performed in a DB-WAX fused silica capillary column (30 m × 0.25 mm i.d., 0.25 μm film thickness, Agilent, Santa Clara, CA, USA) with He as carrier gas (constant linear velocity 36 cm/s). The oven temperature was programmed at 40 °C for 5 min, increased by 5 °C/min to 180 °C, and then by 30 °C/min to 240 °C (and held for 5 min). The mass spectrometer was operated in the electron ionization mode with the electron energy set at 70 eV and 40–300 *m*/*z* scan mass range. Source and interface temperatures were set at 230 °C and 240 °C, respectively. Annotation of the compounds was accomplished by comparing: (i) the retention indices based on the homologous series of n-alkanes (C8-C24, Niles, IL, USA) with those of authentic compounds (when available) and those of NIST14 library (NIST, Gaithersburg, MD, USA), (ii) MS data with those of reference compounds and MS data obtained from the NIST14 library. GCMS solution (ver. 4.30, Shimadzu), AMDIS (ver. 2.72, NIST) and NIST MS Search (ver. 2.2, NIST) software were used in the identification process. The reliability of identification (RID) was set at three levels. A-level: agreement of retention index (RI) and mass spectrum (MS) with those of an authentic compound analyzed under identical experimental conditions; B-level: agreement of retention index (ΔRI < 20) and mass spectrum (match > 900); C-level: at least ΔRI < 20 or mass spectrum similarity match > 800. The percent normalized peak area of volatile components was calculated for each sample and further used in statistical analysis. 

### 2.6. Statistical Analysis

Differences of mean values in microbial counts and sensory score were statistically tested. The data were subjected to Analysis of Variance (ANOVA) followed by Tukey post hoc test using the IBM^®^ SPSS^®^ statistics 19 software (SPSS Inc., Chicago, IL, USA) and a probability level of *p* ≤ 0.05 was considered statistically significant. The volatiles’ data were processed in Metaboanalyst web platform [32] using univariate (Spearman rank correlation testing) and multivariate (OPLS-DA, orthogonal partial least-squares to latent structures discriminant analysis) testing [33,34]. The variables were autoscaled prior to statistical analysis.

## 3. Results

### 3.1. Sensory Evaluation of Fish

At the beginning of fish shelf-life, the general appearance of the whole ice-stored meagre was excellent (Grade 5). It had shiny skin, convex eyes with black pupils and the internal odors as well as the odor of gills were marine. The sensory characteristics deteriorated faster in fish of July than those of January, reaching the minimum acceptability level (Grade 3) three days earlier than the latter. After this point, the skin had slightly lost its brightness and the eyes were flat with translucent pupils. Therefore, the end of shelf-life was defined at D 15 and D 12 for fish of January and July, respectively. 

For the fillets, the appearance and the odor deteriorated faster than the firmness, reaching a Grade 3 of minimum acceptability on D 7 at 4 °C for both January and July samples. After this time point, the fillet had lost its brightness and the odor was neutral to slightly unpleasant. 

### 3.2. Microbiological Changes

**Figure 1:**
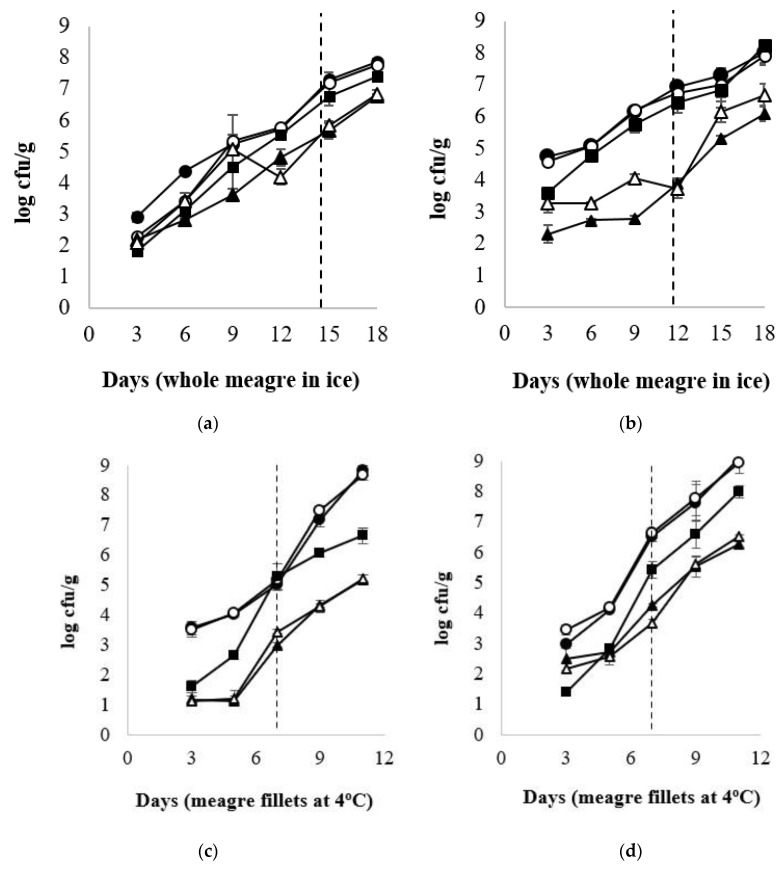
Microbial population changes of whole and filleted chill-stored meagre of January (**a**,**c**) and July (**b**,**d**), respectively. (●) Total Viable Counts (TVC), (o) *Pseudomonas* spp., (■) H_2_S producing bacteria, (▲) LAB—pH 6.4, and (Δ) LAB—pH 8 (●). Each point is the mean of six replicates. Error bars represent the standard error. The dashed vertical line indicates the end of shelf life.

At the beginning of storage (D 3), TVC was 2.90 ± 0.14 log cfu/g and 4.73 ± 0.10 log cfu/g for the whole meagre, and 3.56 ± 0.21 log cfu/g and 2.99 ± 0.11 log cfu/g for the fish fillets, of January and July, respectively (Figure 1). For the whole meagre, microbial populations of TVC, *Pseudomonas* spp., H_2_S-producing bacteria, LAB (pH 6.4) and LAB (pH 8) differed (*p* < 0.05) between both months throughout the storage. More specifically, the microbial populations of fish of July reached higher levels faster than those of fish of January (Figure 1). *Pseudomonas* spp. and H_2_S-producing bacteria were found at higher population levels than the other microorganisms tested throughout the storage (Figure 1). For the fillets, LAB populations (grown on pH 6.4 and pH 8) differed between the two seasons, throughout the storage, in contrast to the H_2_S-producing bacteria counts that were similar for both months (Figure 1). TVC and *Pseudomonas* spp. counts in fish of July were significantly higher than those of January, on the day of minimum acceptability (D 7). H_2_S-producing bacteria had significantly lower counts than *Pseudomonas* spp. in almost all cases (Figure 1).

### 3.3. Microbial Diversity of Chill-Stored Meagre Products

According to HTS analysis, a total of 375,507 raw reads were obtained and after quality checking 269,884 of them were retained, with an average of 16,867 per sample (Table S1). Those high-quality reads were assigned to 936 observed features (range from 28 to 96). The rarefaction to 5000 sequences for estimation of bacterial diversity was quite enough satisfied, since, for example, the Shannon-Wiener Index curves plot (Figure S1) reached a plateau at approximately 500 sequences, indicating a sufficient sequencing depth to characterize microbial diversity.

The results of bacterial diversity at Phylum and Family level are given in Figure S2. Metataxonomic analysis revealed the presence of three main bacterial phyla (Firmicutes, Proteobacteria and Actinobacteria) detected in high relative abundances and other five in traces (Acidobacteria, Spirochaetes, Cyanobacteria, Chlamydiae and Bacteroides), during the whole storage time. In all samples, Proteobacteria (mainly Pseudomonadaceae and to a lesser extent Moraxellaceae) was the most abundant phylum at the end of storage, while at the first stage of storage, this bacterial phylum co-existed with Actinobacteria (mainly Propionibacteriaceae) and Firmicutes (Clostridiaceae). It is crucial to mention that Firmicutes was the most abundant phylum in all but one sample (ArW1_D3) at the first stage of storage.

**Figure 2:**
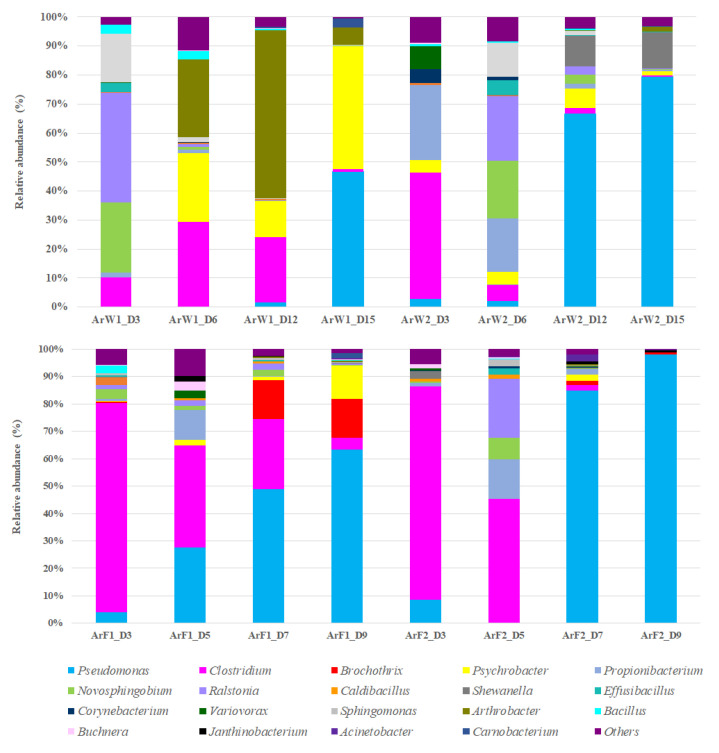
Relative abundance (%) of top-20 bacterial genera of whole (ArW) and filleted (ArF) chill-stored meagre caught in January (1) and July (2), obtained through metabarcoding analysis of 16S rRNA gene at intervals of storage time.

At genus level, *Ralstonia* (37.5%), *Novosphingobium* (24.2%), *Sphingomonas* (16.8%) and *Clostridium* (10.1%) were found initially (D 3) to dominate in the whole meagre of January, while other bacterial genera were found at lower abundances (Figure 2). At advanced period of storage (D 12) the presence of *Arthrobacter* is noteworthy (58%), while at the end of shelf-life (ArW1_D15) potential spoilage bacteria of fish like *Pseudomonas* (46.6%) and *Psychrobacter* (42.5%) clearly dominated. Oppositely, in the whole fish of July, *Clostridium* (43.5%) and *Propionibacterium* (25.9%) were the most abundant bacterial genera at the initial stage (D 3), while at the end of fish shelf-life (ArW2_D12), *Pseudomonas* (66.6%) was found to dominate, followed by *Shewanella* (10.5%). For the fillets, *Pseudomonas* was the most abundant genus at the end of shelf-life (D 7) for both months (Figure 2).

**Figure 3:**
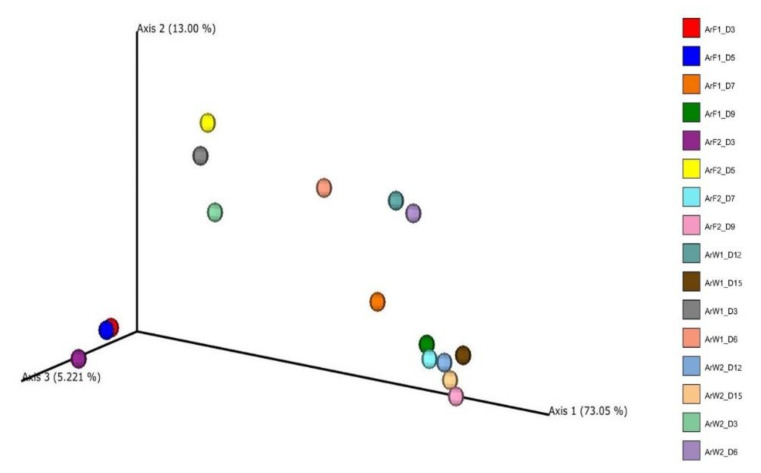
Principal coordinate plot based on weighted UniFrac distance. Different color corresponds to different sample/day. Whole (ArW) and filleted (ArF) chill-stored meagre caught in January (1) and July (2).

Finally, to evaluate potential differences based on samples’ microbiota profile, a PCoA plot was generated based on the weighted UniFrac distance (Figure 3). The analysis indicated a clear separation of samples in advanced storage level and the rejection time point (D 7 for fillets and 12, 15 for whole fish) from the rest. Furthermore, storage temperature (0 and 4 °C for whole and filleted fish, respectively) did not affect samples’ distribution at all. The principal coordinates explained about 91.27% of total variance (vectors 1, 2 and 3 explained 73.05%, 13% and 5.22%, respectively).

### 3.4. Volatile Profile during Fish Storage

A total of 102 volatile compounds were identified in whole and filleted meagre during storage on ice and at 4 °C, respectively (Tables S2 and S3). These included mostly carbonyl compounds (15 aldehydes and 11 ketones), hydrocarbons (21 aliphatic and 8 aromatic) and alcohols (19). A lower number of esters (5), acids (7), terpenoid (11) and miscellaneous compounds (5) were also detected. The volatile profile of both whole and filleted meagre was predominated by alcohols. Their mean content decreased during storage of filleted meagre, whereas the opposite trend was observed in whole iced meagre. The highest content of alcohols was observed at D 12 (73.1%) and D 7 (60.2%) of January for the whole iced and filleted meagre, respectively. Ethanol and 1-penten-3-ol were the most abundant alcohols accounting for up to 50% of the total content of volatiles (Tables S2 and S3). Ketones and aldehydes were the next most abundant chemical classes followed by aliphatic hydrocarbons. The mean content of aldehydes decreased during storage for both whole iced and filleted meagre. On the contrary, the mean content of ketones increased during storage for filleted meagre, whereas the opposite trend was observed for whole iced meagre. Among carbonyl compounds, acetaldehyde, propanal, acetone and acetoin were present at higher levels. The content of aromatic hydrocarbons, esters, acids and terpenoids remained at relatively low levels (<5%) during the storage period for both whole iced and filleted meagre. It is worth to mention the increasing trend of the content of miscellaneous compounds during storage, which was solely due to trimethylamine.

OPLS-DA was carried out to identify volatile compounds showing important variations during the storage of whole and filleted meagre at 0 °C and 4 °C, respectively. Two models were built, one for each case. Both models resulted in one predictive and three orthogonal (1 + 3) components and were found significant when compared with models built on random permutation of labels (Figure S3). As regards the model of whole meagre, the cross-validated predictive ability (Q2Y), the predictor variance (R2X) and the response variance (R2Y) explained by the full model were equal to 91%, 75% and 98%, respectively. As regards the model of filleted meagre, the cross-validated predictive ability (Q2Y), the predictor variance (R2X) and the response variance (R2Y) explained by the full model were equal to 90%, 57% and 99%, respectively.

**Figure 4:**
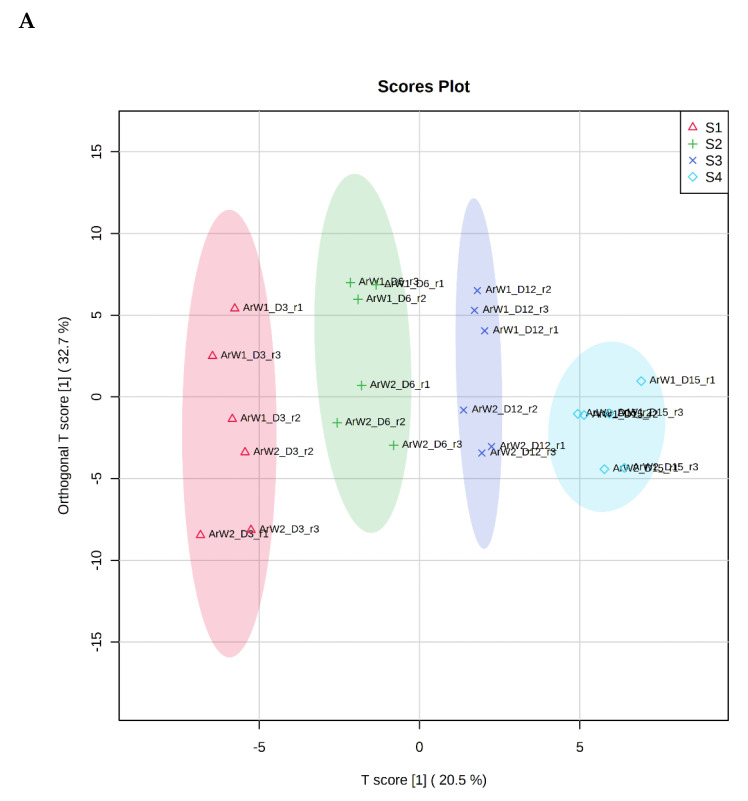

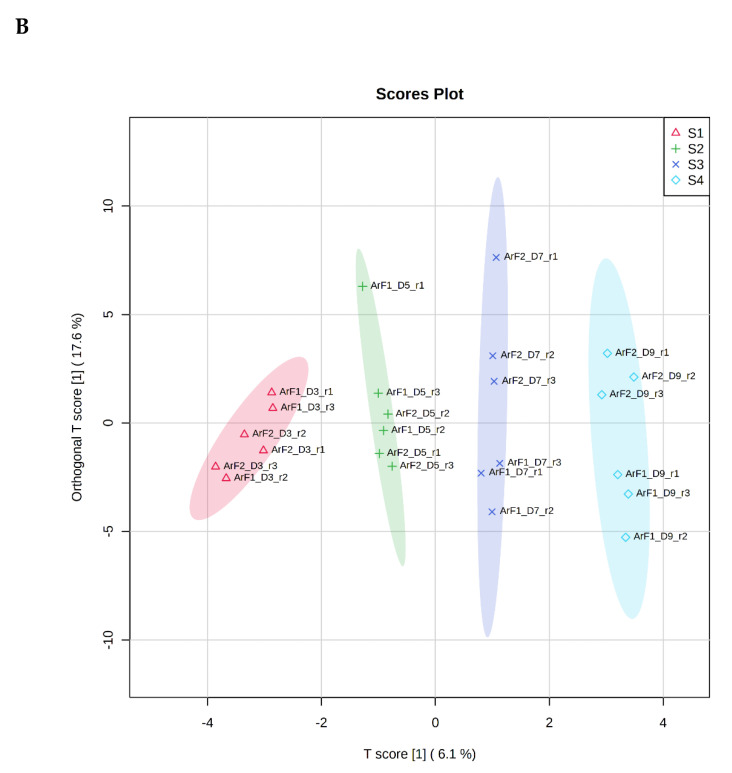
OPLS-DA modeling of volatile compounds’ variations during storage of whole (ArW) and filleted (ArF) meagre caught in January (1) and July (2). Models with one predictive and three orthogonal components were built with the volatile compounds as predictors and the sampling points (S1, S2, S3, S4) as the response. (**A**) Score plot of whole meagre during storage at 0 °C for 3, 6, 12 and 15 days, (**B**) Score plot of filleted meagre during storage at 4 °C for 3, 5, 7 and 9 days. The sampling points S1, S2, S3 and S4 correspond to the increasing days of fish storage. The percentage of the explained response variance is indicated in parentheses. The shaded ellipses correspond to the 95% confidence regions of each class.

It is evident from the score plots (Figure 4) of both models that the four classes (sampling points S1–S4) are separated distinctly. This means that the volatile profile encompasses the biological information about the storage period of either the whole or filleted meagre.

Furthermore, the first orthogonal component indicated two subgroups within each class. This is more apparent in the case of whole meagre (Figure 4A). These subgroups correspond to fish cultured during January and July. Thus, it can be inferred that the within class separation (orthogonal component) depicts the effect of seasonal variation on the volatile profile of meagre. This is further supported by the results of OPLS modeling using as response variable the fish culturing season (Figure S4).

To identify the most interesting volatile compounds showing variations during fish storage, we used a combination of multivariate (VIP) and univariate metrics (pFDR, i.e., *p*-values corrected for multiple testing) from OPLS-DA and Spearman rank correlation test, respectively, according to the methodology described by Thévenot et al. [33]. First, for each variable, *p*-values from the non-parametric hypothesis testing of the correlation with the storage period were computed. A total of 13 volatile compounds were significant (pFDR < 0.05) for the whole meagre, whereas 4 compounds were significant in the case of filleted meagre. Subsequently, those compounds with VIP < 1 were removed from the selection. The final list of the biochemically interesting compounds in whole and filleted meagre is presented in Table 1 (trimethylamine, 3-methylbutanoic acid, 3-methyl-1-butanol, benzaldehyde, heptanal, octanal, 1-butanol, 1-pentanol, ethyl acetate and four unknown alkanes) and Table 2 (trimethylamine, 3-methylbutanoic acid, 3-methyl-1-butanol, 1-pentanol), respectively. Two groups of compounds can be distinguished; one group is represented by compounds whose relative content increases during fish storage and the second group by compounds whose content decreases either gradually during storage or abruptly after 3 days of storage (sampling point S1).

## 4. Discussion

Fish producers, processors, and distributors work towards fisheries and aquaculture products supply to meet consumer demands. Herein, information on sensory, and microbiological changes, and more importantly on microbial communities’ composition and VOCs profile, during chilled storage of the whole and filleted meagre from the Hellenic aquaculture, is highlighted for the first time, to help stakeholders provide products of a high-quality level in national and international commerce. In Greece, there are two more studies on quality characteristics of meagre, such as sensory, somatometric and chemical characteristics e.g., fatty acid profiles and volatile organic compounds, but this information only concerns the raw fish; meaning fish just after harvesting [4,15]. These researchers have proved that meagre is a species that contains fat of high nutritional value and can give high fillet yield (42.2%). All these findings enhance the interest of stakeholders to produce large quantities of this kind of fish, and much more their interest to supply a high-quality food in commerce. 

Shelf-life of fresh fish from the Mediterranean aquaculture, such as gilt-head seabream and European seabass, is usually ranged from 12 to 14 days for the whole fish in ice [13,14], and from 5 to 6 days for their fillets stored at 4 °C [8,17]. For meagre, shelf-life has been found to be 9 days for the whole and filleted fish in ice [6,35]. However, in our case, the whole ice-stored fish was acceptable for human consumption until 12 and 15 days after catch, depending on the season of harvest. Fish shelf-life depends on the season since the mean water temperature in Greece differs approximately 10 or more degrees Celsius, between January and July. It is known that the water temperature can affect the population and composition of the bacteria present in seawater and fish [7,36]. Moreover, the conditions wherein fish remains after catch (temperature in the aquaculture facilities until packaging in ice) and those wherein the products are distributed and marketed (air temperature in distribution and marketing after packaging in ice) strongly differ between winter and summer in Greece. Indeed, the initial TVC differed significantly between the whole meagre taken in January and July. In particular, fish caught in July presented TVC 2 log cfu/g higher than this observed for fish caught in January. Under iced storage conditions, TVC was at higher population levels in fish of July than those of January for the same time points during storage, reaching spoilage population levels of 7–8 log cfu/g three days earlier in fish of July than those of January. In the case of fish fillets, filleting can increase significantly the population levels of bacteria and change the microbial composition compared to the whole fish [8]. This means that the most important factor that determines the shelf-life of meagre fillets (7 days for fish of both months) differs from the factors that mainly affect the shelf-life of the whole meagre.

The findings of the metabarcoding analysis revealed that, at the initial stage of storage, besides the presence of cosmopolitan bacteria belonging to the genera *Propionibacterium*, *Psychrobacter* and *Sphingomonas*, which are commonly found in several fresh seafoods [37,38,39], *Clostridium* was the most abundant bacterial group. However, it is crucial to point out that the microbial communities profile differed in whole fish from the two seasons during the whole storage period. More specifically, *Clostridium* was the most abundant bacteria at D 3 in fish of July, while in fish of January, *Ralstonia* and *Novosphingobium* were found at higher abundances. Several fresh seafoods may be dominated by such microbial groups, as a result of water and/or soil contamination. Indeed, seafood has been characterized as an ideal reservoir of pathogenic microbes that are closely related to human diseases (e.g., via the production of neurotoxin), such as some species belonging to the genus *Clostridium* e.g., *C. botulinum*, *C. perfringens* [40,41], the presence of which, is mainly linked with a potential sewage contamination [42]. Furthermore, *Ralstonia* was found to dominate in the gut of cultured sea bass, Nile Tilapia and Maryland blue crab [43,44,45]. This bacterial group may be responsible for an initial deterioration of sensory attributes, via the production of specific enzymes, closely linked to food/seafood spoilage, such as lipase [46]. Other noteworthy generas, revealed by the present work, such as *Novosphingobium*, were also found at high abundance in fresh Atlantic salmon [47], as well as in the intestine of farmed rainbow trout [48]. This bacterial group is involved in the metabolism of nitrogenous and degradation of aromatic compounds [49,50].

It is well-known that at the late stages of storage, *Pseudomonas* are among the most important spoilers of fresh fish at low temperatures [11,13,51,52,53]. Indeed, based on the 16S metabarcoding analysis, *Pseudomonas* was the dominant bacterial group in almost all samples (whole and filleted fish) at the final stages of storage time, except from sample ArW1_D15, where *Pseudomonas* co-existed with *Psychrobacter*, indicating a microbiota profile-dependent on season of harvest. Other studies examining the dominant spoiler group in fish from the Hellenic seawaters such as farmed gilt-head sea bream confirmed the findings of the present work [23,54]. *Psychrobacter* has also been found to dominate in farmed sea bass [55], rose shrimp [56] and farmed mussels [57]. However, Parlapani et al. [23] noted a geographical-dependent profile of spoiled farmed gilt-head sea bream, where *Psychrobacter* dominated in samples from the Aegean Sea, while *Pseudomonas* was the main spoiler in sea bream from the Ionian waters. *Psychrobacter* and *Pseudomonas* are also part of dominant microbiota of fish from other basins, such as the fillet of chill-stored Atlantic cod [58], while individually *Pseudomonas* or *Psychrobacter* dominate in hake and plaice fillets depending on fish species, storage temperature and lot [39]. Moreover, in the present work, *Shewanella* was found at a remarkable abundance in the whole meagre of July at the end of shelf-life (ArW2_D12), while *Arthrobacter* dominated in the respective time point of storage of fish of January, even though, as previously mentioned, at the end of shelf-life (ArW1_D15), *Pseudomonas* and *Psychrobacter* were the main spoiler players. H_2_S producing bacteria (mainly *Shewanella putrefaciens*) have also been recognized as a key spoiler group of fresh fish at low temperatures [11,13,51,52], while *Arthrobacter* is often noted as a part of the initial microbiota of seafood [59,60] but not as a part of the dominant microbiota at the late stages of storage. To our knowledge, this is the first study that reported this genus as a dominant bacterial group close to the rejection time point of fish. On this point, it should be mentioned the crucial contribution of 16S metataxonomic analysis to unveil the microbiota (both cultivable and non-cultivable) changes during the storage of meagre. This modernized molecular approach surpasses the shortcomings and the limitations of previous conventional methods, constituting an appropriate way to enrich our knowledge on those aspects. The present work accompanies with previous studies on the need of using this method as the most suitable to obtain a comprehensive and reliable snapshot on seafood spoilage microbiota [16,22,23].

The individual compounds identified herein are among those typically observed in fresh marine fish in our previous studies [12,13,14]. Of these, some compounds e.g., trimethylamine, 3-methyl-1-butanol, 3-methylbutanal and ethyl acetate have been reported as bacterial metabolites, while other compounds e.g., 1-octen-3-ol, 1-penten-3-ol, hexanal, nonanal, heptanal, as products of chemical oxidation of fatty acids or other chemical reactions [9]. In other studies, some bacterial metabolites e.g., ethanol, acetic acid, 2-methylbutanal, 3-methylbutanal, 1-propanol, 2-methyl-1-butanol, 3-methyl-1-butanol, 3-hydroxy-2-butanone, 2-butanone, and ethyl esters, were found to increase during fish storage because of *Pseudomonas*, *Shewanella*, LAB, Enterobacteriaceae, *Brochothrix thermosphacta*, *Photobacterium phosphoreum* activity [61,62,63,64]. These compounds have been proposed as potential spoilage markers of fish [13,14,61,63,65,66,67,68,69,70]. In our case, the most interesting compounds (i.e., those with high Spearman’s *p* and VIP), such as trimethylamine, 3-methylbutanoic acid and 3-methyl-1-butanol were found to increase during cold storage of meagre, where *Pseudomonas* and *Psychrobacter* or *Shewanella* dominated. Indeed, these compounds have been linked with metabolic activity of the aforementioned bacteria, mainly of *Pseudomonas*, during storage of fish or inoculated fish model systems in other studies [64,71,72,73,74,75]. It is interesting that trimethylamine was found as a candidate for spoilage marker in meagre, a fish from Greek seawaters. Despite the fact that trimethylamine is produced in large amounts in fish from Northern seas as metabolic product of *Shewanella* and *Photobacterium* [10], it is produced at negligible amounts in fish caught in Mediterranean waters even after long storage [51,76]. This can be ascribed to the low level of precursor compound trimethylamine oxide in fish from such water [51,76,77]. With the exception of aquacultured sea-bass where trimethylamine oxide concentration was 16 mmol/kg [77], there is a lack of such data and it would be interesting to determine trimethylamine oxide content in aquacultured meagre in a future study. 

Regarding compounds associated with chemical activity, no such VOCs were found to increase in the whole or filleted meagre. On the other hand, the compounds with a declining trend, such as alkanes, could be potentially used as indicators of freshness loss. Finally, to the best of our knowledge, no previous work occurs in the volatile profile of meagre during storage to compare our findings. Therefore, further research must be done in the field to enrich our knowledge on such quality characteristics of this much promising fish from the Mediterranean aquaculture.

## 5. Conclusions

The whole ice-stored meagre caught in January and July presented different shelf-life (15 and 12 days, respectively), microbial population profile, bacterial communities’ composition, and VOCs profile, indicating that season of harvesting plays an important role in spoilage status of farmed meagre. *Pseudomonas* and *Psychrobacter* were found to be the potential spoilage organisms of the whole meagre of January, while *Pseudomonas* and *Shewanella* of fish of July. Regarding the fillets, *Pseudomonas* might spoil fish from both months. Of the VOCs detected, trimethylamine, 3-methylbutanoic acid and 3-methyl-1-butanol, linked with the metabolic activity of *Pseudomonas*, *Psychrobacter* and/or *Shewanella* in the literature, could be used as spoilage markers of meagre since they increased during cold storage. No compounds of chemical oxidation or other chemical reaction were found to increase in the whole or filleted meagre, while some compounds like alkanes with a declining trend, could be potentially used as indicators of freshness loss. 

The present work deals with the study of the shelf-life of whole and filleted meagre; an ascending fish species from the Hellenic aquaculture with a potential to become a very popular foodstuff, worldwide. The importance of studying the microbiota changes during the storage period (and more importantly at the end of shelf-life) in combination with the evaluation of VOCs produced by this microbiota, is undeniable, while the use of modernized techniques to unveil the hitherto “unknown profile” of such kind of fish, is of great industrial and scientific interest. The present work provides to the stakeholders the “first picture” on what is happening during meagre storage, highlighting several differences in both fish handling/processing (whole-filleted) and season of capture (January-July), triggering in parallel the need for further research on these topics. Thus, our findings are the first step in the attempt to help stakeholders to supply meagre products of a high-quality level in national and international commerce.

## Figures and Tables

**Table 1:** Volatile compounds correlated significantly ^1^ with the storage period of whole meagre on ice.

Compound	ID ^2^	RI ^3^	Spearman’s *p*	VIP	Boxplot ^4^
Trimethylamine	A	598	0.81	1.76	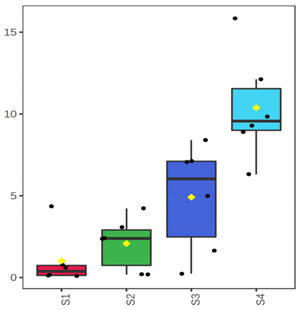
3-Methylbutanoic acid	B	1674	0.70	1.66	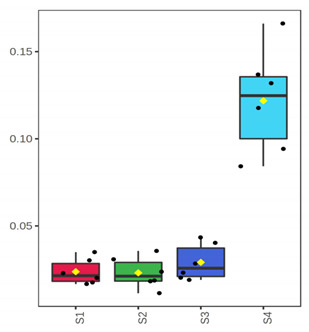
3-Methyl-1-butanol	A	1219	0.61	1.29	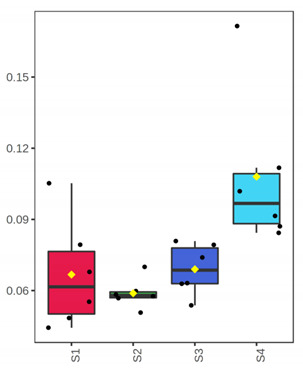
Benzaldehyde	A	1522	−0.86	1.75	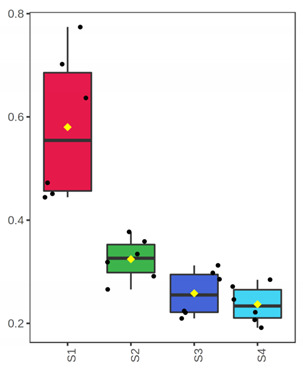
Alkane_1041	-	1041	−0.85	1.81	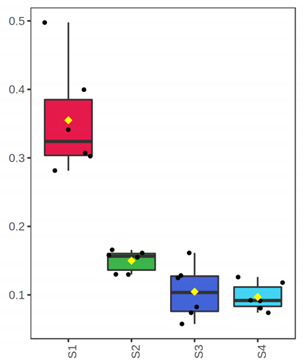
Heptanal	B	1186	−0.82	1.71	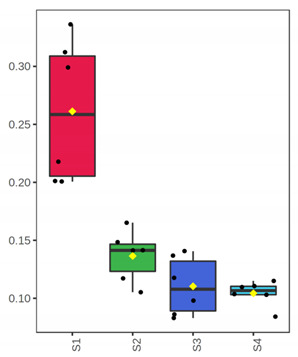
Alkane_1036	-	1036	−0.81	1.74	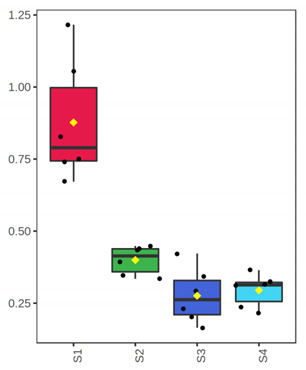
Alkane_1083	-	1083	−0.78	1.71	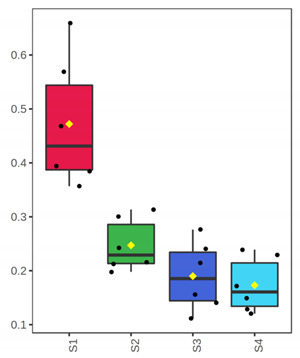
Alkane_1088	-	1088	−0.77	1.77	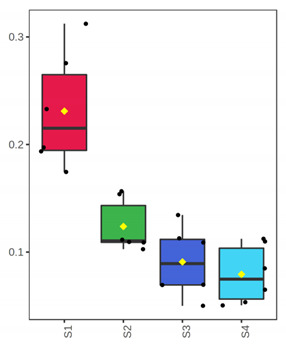
1-Butanol	A	1157	−0.74	1.60	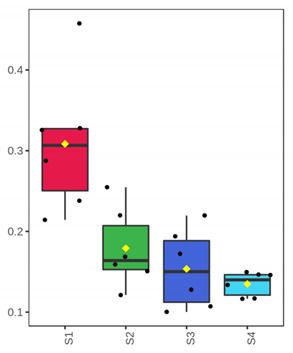
Octanal	A	1290	−0.74	1.62	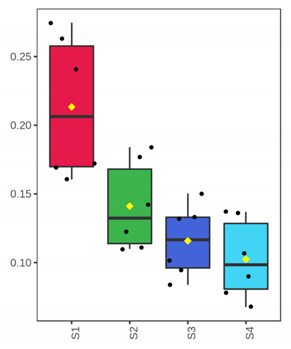
1-Pentanol	A	1261	−0.69	1.56	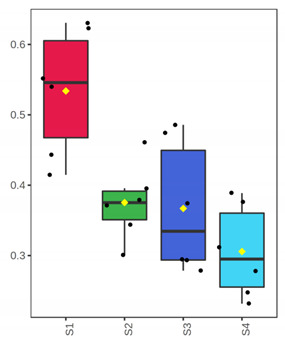
Ethyl acetate	A	884	−0.60	1.53	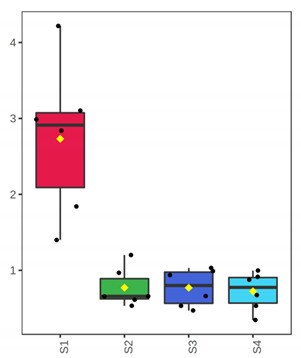

^1^ Correlations were considered significant when pFDR < 0.05 (p-values adjusted for multiple comparison by controlling the false discovery rate at a 5% threshold) and VIP (variable importance in projection) > 1 according to Thévenot et al. [33]. The volatile compounds were sorted by decreasing positive Spearman’s *p* value and then by increasing negative Spearman’s *p* value. ^2^ ID: Identification level (see experimental part). ^3^ RI: Experimental retention index on DB-WAX column. ^4^ The *x*-axis shows the 4 sampling points (S1, S2, S3, S4) which are equivalent to the days of fish storage on ice (3, 6, 12, 15). The *y*-axis shows the percent normalized content.

**Table 2:** Volatile compounds correlated significantly ^1^ with the storage period of filleted meagre at 4 °C.

Compound	ID ^2^	RI ^3^	Spearman’s *p*	VIP	Boxplot ^4^
Trimethylamine	A	598	0.71	2.73	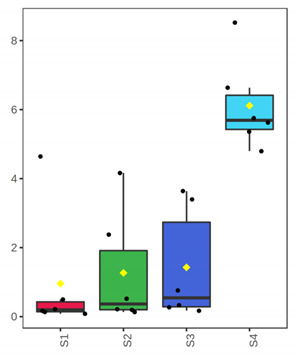
3-Methylbutanoic acid	B	1674	0.73	2.58	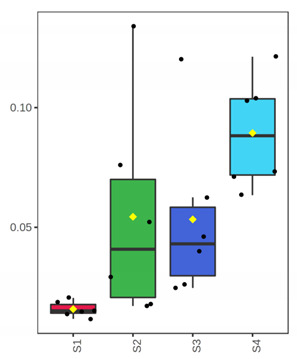
3-Methyl-1-butanol	A	1219	0.60	2.43	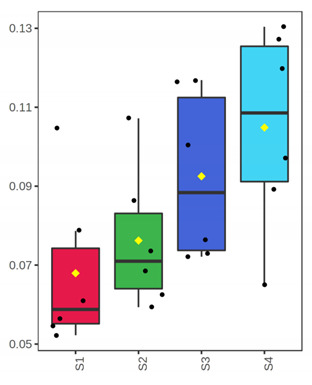
1-Pentanol	A	1261	−0.71	2.38	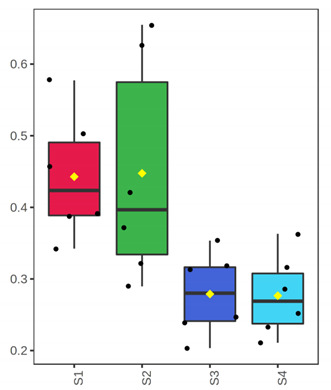

^1^ Correlations were considered significant when pFDR < 0.05 (*p*-values adjusted for multiple comparison by controlling the false discovery rate at a 5% threshold) and VIP (variable importance in projection) > 1 according to Thévenot et al. [33]. The volatile compounds were sorted by decreasing Spearman’s *p* value. ^2^ ID: Identification level (see experimental part). ^3^ RI: Experimental retention index on DB-WAX column. ^4^ The *x*-axis shows the 4 sampling points (S1, S2, S3, S4) which are equivalent to the days of fish storage on ice (3, 5, 7, 9). The *y*-axis shows the percent normalized content.

## Data Availability

The datasets generated for this study are available on request to the corresponding author.

## Supplementary Materials

The following are available online at https://www.mdpi.com/article/10.3390/foods10123109/s1, Table S1: Number of reads and alpha diversity indices of evaluated samples. Whole (ArW) and filleted (ArF) chill-stored meagre caught in January (1) and July (2), Table S2: Relative content (%) of volatile compounds in whole meagre during storage on ice. Values represent the mean content (±standard deviation) of the replicates for each sampling month, Table S3: Relative content (%) of volatile compounds in filleted meagre during storage at 4 °C. Values represent the mean content (±standard deviation) of three replicates for each sampling month, Figure S1: Shannon-Wiener rarefaction curves of whole (ArW) and filleted (ArF) chill-stored meagre caught in January (1) and July (2) at intervals of storage time, obtained through 10 sampling depths, Figure S2: Relative abundance (%) of bacterial phyla (upper) and families (down) of whole (ArW) and filleted (ArF) chill-stored meagre caught in January (1) and July (2), obtained through metabarcoding analysis of 16S rRNA gene at intervals of storage time, Figure S3: Permutation analysis showing the observed and cross-validated R2Y and Q2 coefficients of OPLS-DA model for (A) whole and (B) filleted meagre, Figure S4: Effect of seasonal variation on the volatile compounds of meagre. OPLS-DA score plot of (A) whole meagre caught in January (ArW1) and July (ArW2), (B) filleted meagre caught in January (ArF1) and July (ArF2). The percentage of the explained response variance is indicated in parentheses. The shaded ellipses correspond to the 95% confidence regions of each class.
