# Supplementary material for: Spoilage Investigation of Chill Stored Meagre (Argyrosomus regius) Using Modern Microbiological and Analytical Techniques
Source: Foods. 2021 Dec 15;10(12):3109. doi: 10.3390/foods10123109 (PMC8702202; doi:10.3390/foods10123109)
Supplement: Supplementary file 1 [file foods-10-03109-s001.zip › foods-1480966-supplementary.pdf]

Table S1. Number of reads and alpha diversity indices of evaluated samples. Whole (ArW) and filleted (ArF) chill-stored meagre caught in January (1) and July (2)

Sample	Number of Raw Reads	Number of Filtered Reads	Observed Features	Shannon-Weiner	Faith PD
ArW1_D3	43,502	29,715	65	3.24	35.59
ArW1_D6	14,056	11,148	67	3.85	36.46
ArW1_D12	14,166	11,635	54	2.38	26.47
ArW1_D15	34,888	20,704	42	2.86	9.21
ArW2_D3	11,897	10,717	51	3.09	36.15
ArW2_D6	26,853	19,405	96	4.23	26.16
ArW2_D12	34,019	21,084	84	4.07	10.69
ArW2_D15	24,682	13,220	45	3.14	9.63
ArF1_D3	8,998	7,918	46	1.99	28.61
ArF1_D5	41,112	37,972	74	1.93	49.55
ArF1_D7	20,293	12,917	72	4.49	28.28
ArF1_D9	36,684	24,835	64	4.02	25.49
ArF2_D3	9,000	8,539	31	1.14	23.61
ArF2_D5	15,308	11,528	54	3.16	31.80
ArF2_D7	14,454	10,980	63	4.09	19.10
ArF2_D9	25,595	17,567	28	3.01	4.10

Table S2. Relative content (%) of volatile compounds in whole meagre during storage on ice. Values represent the mean content (± standard deviation) of three replicates for each sampling month.

Compound	January								July							
	Day 3		Day 6		Day 12		Day 15		Day 3		Day 6		Day 12		Day 15	
Ethanol	18.45	1.19	8.32	0.69	13.25	0.31	13.60	2.30	7.95	0.40	8.53	4.28	12.51	3.35	12.20	2.56
1-Propanol	0.06	0.02	0.07	0.04	0.04	0.01	0.09	0.01	0.04	0.00	0.03	0.00	0.03	0.00	0.04	0.00
1-Methoxy-2-propanol	0.16	0.04	0.15	0.06	0.08	0.06	0.11	0.03	0.30	0.10	0.18	0.02	0.21	0.03	0.14	0.01
1-Butanol	0.25	0.04	0.21	0.05	0.11	0.01	0.13	0.01	0.37	0.08	0.15	0.03	0.20	0.02	0.14	0.02
1-Penten-3-ol	27.42	4.72	39.29	5.50	45.19	2.75	34.09	7.34	21.43	3.78	15.61	2.33	24.19	2.67	33.45	2.89
3-Penten-2-ol	0.10	0.03	0.33	0.17	0.32	0.05	0.19	0.11	0.07	0.01	0.05	0.01	0.10	0.02	0.17	0.01
3-Methyl-1-butanol	0.05	0.01	0.06	0.00	0.07	0.01	0.12	0.04	0.08	0.02	0.06	0.01	0.07	0.01	0.09	0.02
1-Pentanol	0.47	0.07	0.34	0.04	0.29	0.01	0.25	0.02	0.60	0.04	0.41	0.05	0.44	0.06	0.36	0.04
(E)-2-Penten-1-ol	0.19	0.07	0.49	0.20	0.51	0.07	0.27	0.11	0.17	0.03	0.12	0.03	0.18	0.02	0.22	0.02
(Z)-2-Penten-1-ol	3.46	1.42	8.16	3.48	9.72	1.48	4.15	1.69	2.61	0.56	1.61	0.48	2.93	0.58	4.15	0.90
3,4-Hexanediol	0.56	0.04	0.33	0.17	0.26	0.04	0.43	0.06	0.52	0.09	0.24	0.16	0.19	0.07	0.22	0.06
(Z)-3-Hexen-1-ol	0.01	0.00	0.02	0.01	0.03	0.00	0.02	0.01	0.01	0.00	0.01	0.00	0.01	0.00	0.02	0.00
2-Butoxy-ethanol	0.37	0.05	0.27	0.01	0.28	0.03	0.23	0.05	0.67	0.14	0.52	0.04	0.41	0.02	0.41	0.06
1-Octen-3-ol	0.74	0.20	0.76	0.19	0.75	0.12	1.20	0.14	0.67	0.09	0.60	0.25	0.61	0.19	0.70	0.12
1-Heptanol	0.10	0.01	0.09	0.01	0.08	0.01	0.05	0.01	0.15	0.02	0.13	0.02	0.11	0.01	0.10	0.01
2-Ethyl-1-hexanol	2.48	0.03	1.83	0.17	1.58	0.29	1.09	0.21	3.98	0.69	3.51	0.48	2.65	0.20	2.59	0.43
1-Octanol	0.05	0.00	0.04	0.01	0.04	0.01	0.03	0.01	0.07	0.01	0.07	0.01	0.06	0.00	0.06	0.01
2,7-Octadien-1-ol	0.28	0.13	0.39	0.01	0.38	0.02	0.31	0.03	0.27	0.04	0.21	0.06	0.23	0.05	0.28	0.05
Benzyl alcohol	0.04	0.00	0.03	0.00	0.08	0.01	0.04	0.01	0.06	0.01	0.05	0.01	0.05	0.00	0.05	0.01
Sum of Alcohols	55.22	5.08	61.16	6.56	73.08	3.16	56.39	7.88	40.02	3.91	32.10	4.93	45.20	4.33	55.40	4.00
Acetaldehyde	5.65	1.04	4.46	1.26	3.95	0.47	4.79	0.71	3.93	0.15	2.71	0.69	3.21	0.24	2.65	0.99
Propanal	3.33	0.45	4.00	2.33	2.17	0.17	2.63	0.73	4.11	0.80	3.36	0.16	3.62	0.37	2.53	0.66
Butanal	0.40	0.04	0.48	0.21	0.31	0.01	0.40	0.05	0.34	0.05	0.33	0.04	0.41	0.03	0.30	0.09

Compound	January								July							
	Day 3		Day 6		Day 12		Day 15		Day 3		Day 6		Day 12		Day 15	
2-Methylbutanal	0.07	0.01	0.07	0.02	0.05	0.01	0.18	0.05	0.13	0.03	0.10	0.01	0.13	0.03	0.10	0.00
3-Methylbutanal	0.10	0.02	0.12	0.02	0.09	0.02	0.33	0.10	0.17	0.02	0.14	0.01	0.19	0.04	0.16	0.00
Pentanal	0.38	0.12	0.39	0.11	0.27	0.01	0.31	0.02	0.21	0.01	0.19	0.02	0.28	0.02	0.22	0.06
Hexanal	1.80	0.37	1.80	0.74	1.08	0.04	1.53	0.21	1.77	0.22	1.59	0.16	1.66	0.26	1.29	0.17
(E)-2-Pentenal	0.05	0.02	0.09	0.01	0.06	0.01	0.06	0.01	0.04	0.00	0.03	0.01	0.04	0.00	0.03	0.00
Heptanal	0.21	0.01	0.12	0.02	0.09	0.01	0.11	0.00	0.32	0.02	0.15	0.01	0.13	0.01	0.10	0.02
(E)-2-Hexenal	0.04	0.01	0.06	0.02	0.04	0.00	0.05	0.00	0.03	0.01	0.03	0.01	0.03	0.01	0.03	0.00
Octanal	0.17	0.01	0.11	0.01	0.09	0.01	0.08	0.01	0.26	0.02	0.17	0.02	0.14	0.01	0.13	0.02
Nonanal	0.09	0.01	0.09	0.00	0.09	0.01	0.07	0.01	0.14	0.02	0.16	0.03	0.15	0.02	0.14	0.02
2,4-Heptadienal_isomer 1	0.26	0.15	0.23	0.09	0.17	0.04	0.19	0.05	0.13	0.04	0.09	0.05	0.11	0.02	0.10	0.02
2,4-Heptadienal_isomer 2	0.14	0.09	0.16	0.04	0.13	0.04	0.11	0.01	0.11	0.04	0.08	0.04	0.09	0.02	0.09	0.02
Benzaldehyde	0.46	0.01	0.30	0.03	0.22	0.01	0.21	0.01	0.70	0.07	0.35	0.03	0.30	0.01	0.27	0.02
Sum of Aldehydes	13.13	1.21	12.48	2.77	8.82	0.50	11.05	1.05	12.40	0.85	9.48	0.73	10.49	0.51	8.14	1.21
Acetone	8.95	3.43	12.36	0.83	4.51	0.26	4.90	0.93	15.26	3.02	40.04	2.79	20.13	1.21	10.04	0.14
2-Butanone	0.32	0.20	0.26	0.02	0.08	0.01	0.11	0.02	0.20	0.02	0.16	0.05	0.23	0.02	0.23	0.02
2,3-Butanedione	0.22	0.02	0.17	0.05	0.13	0.00	0.23	0.02	0.18	0.01	0.13	0.01	0.15	0.02	0.19	0.01
2,3-Pentanedione	0.91	0.35	1.25	0.66	0.51	0.02	0.60	0.15	0.76	0.16	0.65	0.09	0.71	0.07	0.56	0.06
2,3-Hexanedione	0.12	0.04	0.15	0.04	0.08	0.01	0.08	0.02	0.08	0.01	0.07	0.01	0.08	0.01	0.07	0.01
Acetoin	4.01	0.90	0.78	0.38	0.92	0.19	1.39	0.12	3.58	0.62	1.39	0.46	1.16	0.23	1.94	0.56
2,3-Octanedione	0.04	0.02	0.06	0.02	0.04	0.01	0.03	0.00	0.06	0.01	0.06	0.01	0.05	0.02	0.05	0.01
5-Hepten-2-one, 6-methyl-	0.03	0.01	0.03	0.00	0.02	0.00	0.03	0.00	0.03	0.00	0.04	0.01	0.05	0.01	0.04	0.01
3,5-Octadien-2-one_isomer 1	0.17	0.05	0.26	0.11	0.17	0.03	0.32	0.05	0.12	0.03	0.11	0.05	0.11	0.05	0.11	0.03
3,5-Octadien-2-one_isomer 2	0.04	0.01	0.06	0.01	0.05	0.01	0.08	0.01	0.04	0.01	0.03	0.01	0.04	0.01	0.03	0.01
Acetophenone	0.12	0.01	0.09	0.01	0.10	0.01	0.11	0.02	0.19	0.02	0.21	0.06	0.16	0.01	0.18	0.04
Sum of Ketones	14.93	3.57	15.46	1.13	6.62	0.33	7.88	0.96	20.50	3.09	42.89	2.83	22.88	1.23	13.45	0.58

Compound	January								July							
	Day 3		Day 6		Day 12		Day 15		Day 3		Day 6		Day 12		Day 15	
Hexane	0.13	0.07	0.09	0.04	0.06	0.01	0.09	0.03	0.21	0.03	0.17	0.05	0.14	0.02	0.15	0.03
Octane	0.01	0.00	0.01	0.01	0.00	0.00	0.02	0.01	0.02	0.00	0.01	0.00	0.01	0.00	0.01	0.00
Heptane, 2,4-dimethyl-	0.60	0.24	0.35	0.31	0.06	0.01	0.46	0.15	0.67	0.25	0.05	0.01	0.21	0.05	0.11	0.05
2,4-Octadiene_isomer 1	0.02	0.01	0.02	0.01	0.01	0.01	0.05	0.02	0.01	0.00	0.00	0.00	0.01	0.00	0.01	0.00
2,4-Octadiene_isomer 2	0.02	0.01	0.01	0.01	0.01	0.00	0.03	0.01	0.01	0.00	0.00	0.00	0.01	0.00	0.01	0.00
Heptane, 2,2,4,6,6-pentamethyl-	1.11	0.36	0.63	0.48	0.17	0.03	0.89	0.12	1.70	0.30	0.74	0.12	1.30	0.31	0.81	0.14
Decane	0.02	0.01	0.01	0.01	0.01	0.00	0.03	0.01	0.03	0.01	0.01	0.00	0.02	0.00	0.02	0.00
Alkane_1011	0.34	0.04	0.22	0.08	0.10	0.02	0.19	0.03	0.40	0.12	0.14	0.01	0.19	0.02	0.15	0.04
Alkane_1036	0.75	0.08	0.39	0.04	0.20	0.03	0.29	0.05	1.00	0.24	0.41	0.06	0.35	0.07	0.30	0.08
Alkane_1041	0.31	0.03	0.15	0.02	0.07	0.01	0.09	0.01	0.40	0.10	0.15	0.02	0.14	0.02	0.11	0.02
Alkane_1083	0.38	0.02	0.21	0.01	0.14	0.02	0.13	0.01	0.57	0.10	0.29	0.04	0.24	0.03	0.21	0.04
Alkane_1088	0.19	0.01	0.11	0.00	0.06	0.01	0.06	0.01	0.27	0.04	0.14	0.03	0.12	0.01	0.10	0.02
Undecane	0.01	0.00	0.01	0.00	0.00	0.00	0.01	0.00	0.02	0.00	0.01	0.00	0.01	0.00	0.01	0.00
Dodecane	0.51	0.07	0.34	0.06	0.39	0.06	0.58	0.05	1.14	0.27	0.56	0.03	0.80	0.13	0.66	0.17
Alkane_1250	0.11	0.01	0.10	0.02	0.08	0.02	0.05	0.01	0.14	0.05	0.14	0.01	0.10	0.01	0.11	0.02
Tridecane	0.10	0.02	0.08	0.01	0.08	0.01	0.08	0.01	0.19	0.04	0.11	0.02	0.13	0.02	0.14	0.03
Tetradecane	0.04	0.01	0.04	0.01	0.04	0.00	0.04	0.01	0.08	0.02	0.05	0.01	0.05	0.01	0.06	0.01
Alkane_1490	2.01	0.66	2.07	0.35	2.38	0.25	2.90	0.27	1.45	0.20	1.17	0.47	1.21	0.36	1.51	0.27
Pentadecane	0.13	0.03	0.11	0.02	0.12	0.01	0.12	0.02	0.48	0.15	0.25	0.08	0.33	0.04	0.30	0.07
Pentadecane, 2,6,10,14-tetramethyl-	1.31	0.15	0.97	0.17	1.68	0.09	1.15	0.08	2.02	0.42	2.11	0.62	2.13	0.25	2.32	0.46
Heptadecane	0.38	0.06	0.33	0.05	0.38	0.03	0.35	0.05	0.67	0.17	0.69	0.22	0.65	0.08	0.64	0.09
Sum of Aliphatic Hydrocarbons	8.48	0.82	6.24	0.70	6.03	0.28	7.62	0.36	11.49	0.77	7.19	0.83	8.16	0.57	7.73	0.60
Benzene	0.17	0.02	0.09	0.04	0.09	0.01	0.62	0.12	0.33	0.07	0.09	0.00	0.09	0.01	0.61	0.53
Toluene	0.88	0.08	0.20	0.09	0.21	0.05	0.27	0.06	1.67	0.33	0.25	0.02	0.26	0.03	0.26	0.06
Ethylbenzene	0.28	0.02	0.08	0.03	0.07	0.01	0.13	0.02	0.60	0.09	0.13	0.01	0.14	0.02	0.16	0.03

Compound	January								July							
	Day 3		Day 6		Day 12		Day 15		Day 3		Day 6		Day 12		Day 15	
p-Xylene	0.12	0.01	0.04	0.02	0.03	0.01	0.06	0.01	0.27	0.05	0.05	0.00	0.06	0.01	0.06	0.01
m-Xylene	0.39	0.03	0.12	0.05	0.11	0.02	0.18	0.03	0.88	0.15	0.17	0.01	0.19	0.02	0.19	0.03
o-Xylene	0.22	0.02	0.07	0.02	0.06	0.01	0.10	0.02	0.48	0.07	0.10	0.01	0.11	0.01	0.12	0.02
Styrene	0.14	0.01	0.02	0.00	0.02	0.00	0.03	0.00	0.30	0.05	0.07	0.01	0.05	0.01	0.08	0.02
Naphthalene	0.08	0.01	0.05	0.00	0.04	0.01	0.04	0.01	0.11	0.01	0.08	0.02	0.08	0.01	0.07	0.01
Sum of Aromatic Hydrocarbons	2.28	0.09	0.67	0.12	0.64	0.06	1.44	0.14	4.65	0.39	0.94	0.03	0.98	0.05	1.54	0.54
Methyl Acetate	0.09	0.04	0.01	0.00	0.01	0.00	0.02	0.01	0.13	0.03	0.02	0.01	0.03	0.00	0.05	0.03
Ethyl Acetate	2.03	0.74	0.60	0.06	0.56	0.10	0.52	0.16	3.44	0.68	0.94	0.27	0.99	0.05	0.93	0.06
3-Methylbutyl acetate	0.03	0.01	0.02	0.00	0.02	0.00	0.10	0.01	0.04	0.00	0.04	0.01	0.04	0.00	0.05	0.04
Ethyl 2-hydroxypropanoate	0.27	0.02	0.18	0.00	0.26	0.01	0.28	0.09	0.27	0.01	0.27	0.05	0.24	0.01	0.21	0.03
Ethyl decanoate	0.02	0.00	0.01	0.00	0.02	0.01	0.03	0.01	0.01	0.00	0.00	0.00	0.01	0.00	0.01	0.01
Sum of Esters	2.45	0.74	0.83	0.06	0.87	0.10	0.96	0.19	3.89	0.68	1.28	0.28	1.29	0.05	1.24	0.08
Acetic acid	0.31	0.14	0.14	0.00	0.15	0.05	0.39	0.25	0.59	0.24	0.30	0.03	0.41	0.11	0.43	0.09
Propanoic acid	0.03	0.00	0.02	0.01	0.02	0.00	0.03	0.01	0.04	0.01	0.03	0.01	0.02	0.00	0.02	0.00
Butanoic acid	0.13	0.03	0.07	0.01	0.05	0.01	0.27	0.14	0.43	0.19	0.20	0.07	0.16	0.06	0.18	0.06
3-Methylbutanoic acid	0.03	0.01	0.03	0.01	0.02	0.00	0.11	0.02	0.02	0.00	0.02	0.01	0.03	0.01	0.13	0.04
Hexanoic acid	0.08	0.03	0.06	0.02	0.05	0.00	0.12	0.04	0.14	0.03	0.14	0.05	0.08	0.02	0.13	0.05
Octanoic acid	0.03	0.03	0.02	0.00	0.03	0.02	0.02	0.00	0.04	0.01	0.03	0.01	0.02	0.01	0.05	0.02
Nonanoic acid	0.06	0.05	0.03	0.00	0.09	0.06	0.03	0.00	0.06	0.00	0.05	0.01	0.04	0.00	0.07	0.03
Sum of Acids	0.67	0.16	0.37	0.03	0.42	0.08	0.99	0.29	1.32	0.31	0.77	0.10	0.77	0.13	1.00	0.13
a-Pinene	0.66	0.21	0.38	0.32	0.13	0.01	0.58	0.18	0.83	0.09	0.24	0.03	0.44	0.10	0.24	0.05
b-Pinene	0.06	0.01	0.03	0.02	0.01	0.00	0.04	0.01	0.11	0.01	0.03	0.00	0.04	0.01	0.03	0.01
Thuja-2,4(10)-diene	0.08	0.02	0.01	0.01	0.01	0.00	0.03	0.01	0.12	0.01	0.02	0.00	0.02	0.00	0.01	0.00
3-Carene	0.05	0.01	0.02	0.01	0.02	0.00	0.03	0.01	0.09	0.01	0.03	0.00	0.04	0.00	0.03	0.01
b-Myrcene	0.04	0.01	0.03	0.01	0.02	0.00	0.04	0.01	0.07	0.01	0.03	0.00	0.05	0.01	0.04	0.01

Compound	January								July							
	Day 3		Day 6		Day 12		Day 15		Day 3		Day 6		Day 12		Day 15	
D-Limonene	0.70	0.21	0.61	0.06	0.50	0.06	0.60	0.12	1.00	0.13	0.89	0.19	1.06	0.05	0.85	0.13
g-Terpinene	0.12	0.04	0.09	0.07	0.04	0.01	0.15	0.05	0.16	0.02	0.04	0.01	0.09	0.02	0.06	0.01
p-Cymene	0.42	0.08	0.32	0.23	0.18	0.03	0.51	0.17	0.84	0.10	0.21	0.04	0.38	0.09	0.31	0.08
Terpene_alcohol_1476	0.03	0.01	0.02	0.01	0.04	0.02	0.06	0.02	0.04	0.00	0.05	0.01	0.05	0.01	0.08	0.05
b-Gurjunene	0.14	0.02	0.09	0.01	0.09	0.01	0.10	0.01	0.25	0.04	0.17	0.04	0.20	0.03	0.18	0.03
Calamenene	0.03	0.01	0.02	0.01	0.02	0.00	0.02	0.00	0.05	0.01	0.05	0.01	0.05	0.01	0.05	0.00
Sum of Terpenoids	2.32	0.31	1.64	0.41	1.05	0.07	2.16	0.28	3.57	0.19	1.76	0.20	2.41	0.15	1.88	0.17
Trimethylamine	0.27	0.29	0.92	1.26	2.29	2.44	11.35	3.90	1.77	2.26	3.24	0.92	7.53	0.76	9.42	2.92
Dimethyl sulfide	0.02	0.01	0.02	0.00	0.01	0.00	0.01	0.00	0.08	0.01	0.05	0.01	0.04	0.01	0.04	0.01
2-Ethylfuran	0.09	0.04	0.11	0.05	0.05	0.01	0.09	0.02	0.09	0.01	0.07	0.01	0.07	0.01	0.05	0.01
2-Pentylfuran	0.09	0.01	0.03	0.01	0.02	0.00	0.04	0.01	0.17	0.02	0.04	0.00	0.04	0.00	0.03	0.01
2,4,6-Trimethyl-pyridine	0.06	0.01	0.07	0.05	0.09	0.07	0.04	0.03	0.06	0.05	0.20	0.02	0.13	0.01	0.06	0.02
Sum of Miscellaneous	0.53	0.29	1.16	1.26	2.47	2.44	11.52	3.90	2.16	2.26	3.59	0.92	7.81	0.76	9.62	2.92

Table S3. Relative content (%) of volatile compounds in filleted meagre during storage at 4 °C. Values represent the mean content (± standard deviation) of three replicates for each sampling month.

Compound	January								July							
	Day 3		Day 5		Day 7		Day 9		Day 3		Day 5		Day 7		Day 9	
2-Methyl-2-propanol	0.12	0.05	0.19	0.05	0.15	0.02	0.16	0.01	0.14	0.06	0.12	0.15	0.35	0.22	0.14	0.01
Ethanol	22.57	9.46	15.70	2.54	17.50	3.65	12.97	3.84	20.40	1.86	5.64	2.51	14.53	0.78	8.17	2.16
1-Propanol	0.03	0.01	0.02	0.00	0.02	0.00	0.02	0.00	0.02	0.00	0.02	0.00	0.02	0.00	0.02	0.00
1-Methoxy-2-propanol	0.16	0.05	0.25	0.09	0.21	0.04	0.10	0.02	0.25	0.10	0.60	0.24	0.44	0.04	0.26	0.10
1-Butanol	0.14	0.01	0.61	0.20	0.63	0.16	0.29	0.13	0.43	0.04	0.50	0.18	1.07	0.55	0.40	0.13
1-Penten-3-ol	27.02	2.78	25.16	6.75	34.23	0.66	34.08	7.49	28.42	3.10	34.57	6.61	16.51	1.84	24.38	4.04
3-Penten-2-ol	0.06	0.02	0.05	0.02	0.05	0.00	0.07	0.01	0.06	0.01	0.05	0.01	0.02	0.00	0.05	0.01
3-Methyl-1-butanol	0.05	0.00	0.07	0.01	0.07	0.00	0.10	0.03	0.08	0.02	0.09	0.02	0.11	0.01	0.11	0.02
1-Pentanol	0.49	0.10	0.32	0.04	0.23	0.02	0.23	0.02	0.40	0.06	0.42	0.13	0.33	0.02	0.32	0.04
(E)-2-Penten-1-ol	0.25	0.06	0.18	0.09	0.19	0.01	0.27	0.02	0.25	0.04	0.18	0.01	0.14	0.00	0.20	0.05
(Z)-2-Penten-1-ol	3.31	0.10	3.06	2.02	4.24	0.72	5.19	0.60	3.80	0.59	4.36	0.95	1.65	0.24	2.73	0.81
3,4-Hexanediol	0.37	0.11	0.38	0.06	0.26	0.08	0.32	0.10	0.19	0.05	0.09	0.05	0.10	0.01	0.22	0.07
(Z)3-Hexen-1-ol	0.05	0.01	0.04	0.00	0.04	0.00	0.04	0.00	0.18	0.03	0.17	0.09	0.11	0.01	0.08	0.01
2-Butoxy-ethanol	0.53	0.08	0.54	0.05	0.65	0.10	0.38	0.02	0.77	0.20	0.73	0.14	1.29	0.11	0.79	0.12
1-Octen-3-ol	0.66	0.14	0.80	0.18	0.54	0.05	0.84	0.10	0.59	0.14	0.61	0.18	0.45	0.06	0.89	0.28
1-Heptanol	0.03	0.00	0.03	0.00	0.03	0.00	0.02	0.00	0.03	0.03	0.04	0.10	0.05	0.00	0.04	0.01
2-Ethyl-1-hexanol	0.61	0.08	0.72	0.07	0.84	0.12	0.45	0.04	0.77	0.10	0.86	2.03	1.05	0.16	0.72	0.10
1-Octanol	0.07	0.01	0.05	0.01	0.07	0.01	0.05	0.00	0.09	0.02	0.09	0.01	0.11	0.02	0.08	0.01
2,3-Butanediol	1.16	0.95	1.74	0.74	0.17	0.04	0.12	0.05	0.12	0.04	0.09	0.18	0.13	0.02	0.10	0.03
2,7-Octadien-1-ol	0.01	0.01	0.01	0.00	0.03	0.02	0.01	0.00	0.00	0.00	0.00	0.01	0.05	0.05	0.01	0.00
Benzyl alcohol	0.05	0.01	0.08	0.01	0.07	0.01	0.05	0.00	0.09	0.01	0.09	0.02	0.09	0.01	0.07	0.01
Sum of Alcohols	57.73	9.91	50.01	7.54	60.22	3.78	55.76	8.44	57.08	3.67	49.34	7.43	38.60	2.11	39.76	4.67
Acetaldehyde	6.38	0.42	6.16	0.58	6.06	1.40	5.71	0.37	4.93	0.40	4.11	0.06	4.26	0.11	3.73	0.27

Compound	January								July							
	Day 3		Day 5		Day 7		Day 9		Day 3		Day 5		Day 7		Day 9	
Propanal	4.14	2.17	3.41	0.36	2.76	0.76	2.53	0.68	3.98	0.62	3.97	0.46	4.75	0.02	4.67	0.42
Butanal	0.41	0.18	0.49	0.03	0.36	0.05	0.37	0.11	0.33	0.06	0.38	0.04	0.34	0.03	0.47	0.03
2-Methylbutanal	0.04	0.01	0.09	0.00	0.05	0.02	0.08	0.02	0.04	0.02	0.09	0.01	0.07	0.02	0.08	0.00
3-Methylbutanal	0.05	0.01	0.16	0.01	0.11	0.02	0.15	0.03	0.08	0.03	0.15	0.02	0.08	0.01	0.14	0.03
Pentanal	0.27	0.06	0.31	0.03	0.21	0.03	0.23	0.02	0.19	0.03	0.25	0.03	0.18	0.02	0.22	0.00
Hexanal	1.53	0.30	1.31	0.10	0.78	0.07	1.06	0.11	1.49	0.31	1.36	0.42	1.21	0.21	1.40	0.18
(E)-2-Pentenal	0.03	0.01	0.04	0.01	0.02	0.00	0.03	0.01	0.03	0.00	0.03	0.01	0.03	0.00	0.04	0.01
Heptanal	0.07	0.01	0.07	0.00	0.05	0.00	0.06	0.00	0.08	0.01	0.08	0.12	0.08	0.01	0.08	0.01
(E)-2-Hexenal	0.05	0.01	0.06	0.01	0.04	0.00	0.04	0.01	0.15	0.02	0.14	0.06	0.09	0.01	0.09	0.01
Octanal	0.05	0.00	0.05	0.00	0.05	0.01	0.04	0.00	0.06	0.01	0.05	0.13	0.07	0.00	0.06	0.00
Nonanal	0.15	0.03	0.12	0.03	0.15	0.01	0.12	0.01	0.24	0.05	0.27	0.06	0.24	0.03	0.20	0.02
2,4-Heptadienal_isomer 1	0.14	0.02	0.15	0.02	0.11	0.03	0.13	0.02	0.11	0.03	0.11	0.04	0.12	0.02	0.18	0.03
2,4-Heptadienal_isomer 2	0.11	0.03	0.11	0.03	0.07	0.01	0.10	0.02	0.10	0.03	0.08	0.05	0.08	0.01	0.13	0.03
Benzaldehyde	0.14	0.01	0.18	0.03	0.15	0.02	0.17	0.02	0.16	0.01	0.18	0.28	0.18	0.01	0.20	0.00
Sum of Aldehydes	13.57	2.24	12.69	0.70	10.99	1.59	10.82	0.79	11.96	0.80	11.24	0.72	11.77	0.25	11.68	0.53
Acetone	8.94	1.50	12.43	1.78	8.81	1.62	10.70	2.38	11.56	1.54	17.97	2.63	23.21	3.17	16.93	3.22
2-Butanone	0.14	0.03	1.48	0.49	1.10	0.22	0.51	0.13	0.17	0.02	0.32	0.14	0.41	0.03	0.27	0.04
2,3-Butanedione	0.17	0.03	0.18	0.02	0.16	0.04	0.15	0.03	0.17	0.03	0.15	0.02	0.15	0.02	0.23	0.02
2,3-Pentanedione	0.63	0.06	0.61	0.03	0.57	0.05	0.59	0.02	0.55	0.14	0.54	0.20	0.56	0.07	0.72	0.09
2,3-Hexanedione	0.08	0.01	0.09	0.00	0.07	0.01	0.10	0.02	0.05	0.01	0.05	0.03	0.05	0.01	0.06	0.00
Acetoin	2.09	0.38	2.09	0.45	0.98	0.26	0.94	0.10	2.86	0.50	0.87	0.28	0.68	0.11	3.06	0.24
2,3-Octanedione	0.04	0.01	0.04	0.01	0.03	0.00	0.04	0.00	0.06	0.01	0.06	0.02	0.04	0.01	0.05	0.01
6-Methyl-5-hepten-2-one	0.03	0.00	0.04	0.01	0.03	0.00	0.02	0.00	0.04	0.01	0.05	0.01	0.05	0.00	0.05	0.01
3,5-Octadien-2-one_isomer 1	0.15	0.03	0.29	0.06	0.14	0.01	0.24	0.02	0.10	0.03	0.09	0.05	0.09	0.01	0.17	0.05
3,5-Octadien-2-one_isomer 2	0.05	0.01	0.06	0.02	0.04	0.00	0.07	0.01	0.03	0.01	0.03	0.01	0.03	0.00	0.05	0.02

Compound	January								July							
	Day 3		Day 5		Day 7		Day 9		Day 3		Day 5		Day 7		Day 9	
Acetophenone	0.20	0.05	0.30	0.06	0.37	0.03	0.14	0.01	0.10	0.00	0.11	0.04	0.13	0.01	0.10	0.02
Sum of Ketones	12.52	1.55	17.62	1.91	12.29	1.66	13.49	2.39	15.69	1.63	20.22	2.65	25.39	3.18	21.69	3.24
Hexane	0.14	0.01	0.14	0.03	0.14	0.02	0.08	0.01	0.15	0.05	0.15	0.19	0.33	0.02	0.22	0.02
Heptane, 2,4-dimethyl-	0.17	0.08	0.50	0.18	0.11	0.04	0.11	0.01	0.07	0.04	0.05	0.25	0.10	0.03	0.16	0.02
Heptane, 2,2,4,6,6-pentamethyl-	0.59	0.30	1.63	0.69	0.47	0.14	0.27	0.04	0.34	0.17	0.51	0.10	2.56	0.57	1.64	0.40
Decane	0.39	0.15	0.70	0.27	0.29	0.07	0.16	0.01	0.23	0.08	0.31	0.14	1.09	0.21	0.77	0.14
Alkane_1011	0.26	0.09	0.38	0.15	0.18	0.04	0.10	0.02	0.10	0.03	0.13	0.11	0.32	0.04	0.26	0.04
Alkane_1036	0.29	0.09	0.30	0.12	0.19	0.02	0.11	0.02	0.06	0.02	0.08	0.33	0.09	0.01	0.13	0.05
Alkane_1041	0.12	0.04	0.13	0.05	0.08	0.01	0.04	0.01	0.06	0.02	0.08	0.09	0.03	0.00	0.04	0.02
Alkane_1083	0.17	0.03	0.14	0.05	0.13	0.01	0.07	0.01	0.06	0.01	0.07	0.31	0.08	0.01	0.09	0.03
Alkane_1088	0.09	0.01	0.07	0.03	0.07	0.00	0.04	0.01	0.03	0.01	0.03	0.16	0.03	0.01	0.04	0.01
Undecane	0.22	0.02	0.31	0.08	0.18	0.02	0.12	0.01	0.20	0.04	0.25	0.03	0.46	0.02	0.36	0.05
Dodecane	0.64	0.05	0.79	0.23	0.55	0.10	0.34	0.02	0.49	0.11	0.72	0.09	1.43	0.17	1.11	0.19
Alkane_1250	0.32	0.03	0.15	0.04	0.28	0.04	0.22	0.02	0.33	0.05	0.35	0.11	0.29	0.06	0.25	0.06
Tridecane	0.27	0.03	0.21	0.01	0.23	0.04	0.18	0.01	0.23	0.03	0.26	0.06	0.31	0.04	0.27	0.02
Tetradecane	0.09	0.01	0.07	0.01	0.09	0.02	0.07	0.00	0.10	0.02	0.09	0.01	0.11	0.02	0.10	0.01
Alkane_1490	1.94	0.12	2.24	0.64	1.86	0.14	2.37	0.16	1.43	0.24	1.98	0.36	1.06	0.18	2.03	0.55
Pentadecane	0.21	0.02	0.29	0.04	0.21	0.02	0.13	0.02	0.30	0.09	0.38	0.09	0.52	0.06	0.43	0.02
Pentadecane, 2,6,10,14-tetramethyl-	1.48	0.08	1.75	0.40	1.69	0.40	1.23	0.08	1.94	0.37	2.76	1.18	2.35	0.76	2.54	0.60
Heptadecane	0.39	0.02	0.55	0.01	0.47	0.03	0.33	0.04	0.52	0.13	0.64	0.26	0.78	0.26	0.73	0.26
Sum of Aliphatic Hydrocarbons	7.79	0.41	10.35	1.12	7.21	0.47	5.96	0.20	6.63	0.53	8.85	1.41	11.96	1.05	11.15	0.99
Benzene	0.18	0.03	0.17	0.02	0.15	0.02	0.10	0.01	0.07	0.06	0.11	0.10	0.16	0.01	0.15	0.03
Toluene	0.54	0.08	1.34	0.39	0.69	0.08	0.36	0.03	0.27	0.07	0.27	0.29	0.68	0.14	0.45	0.09
Ethylbenzene	0.21	0.02	0.38	0.11	0.17	0.02	0.13	0.01	0.16	0.04	0.20	0.07	0.61	0.21	0.36	0.11
p-Xylene	0.08	0.01	0.10	0.03	0.06	0.01	0.04	0.00	0.09	0.03	0.08	0.02	0.13	0.03	0.08	0.01

Compound	January								July							
	Day 3		Day 5		Day 7		Day 9		Day 3		Day 5		Day 7		Day 9	
m-Xylene	0.24	0.01	0.32	0.09	0.20	0.03	0.13	0.00	0.23	0.06	0.23	0.08	0.44	0.09	0.28	0.06
o-Xylene	0.15	0.01	0.20	0.06	0.12	0.02	0.09	0.00	0.14	0.03	0.15	0.03	0.30	0.06	0.19	0.04
Styrene	0.11	0.03	0.36	0.13	0.11	0.02	0.07	0.01	0.06	0.01	0.16	0.07	0.71	0.26	0.45	0.16
Naphthalene	0.07	0.01	0.08	0.01	0.07	0.00	0.05	0.00	0.08	0.01	0.10	0.02	0.11	0.01	0.09	0.01
Sum of Aromatic Hydrocarbons	1.58	0.09	2.94	0.45	1.57	0.10	0.98	0.04	1.09	0.12	1.31	0.34	3.15	0.37	2.05	0.23
Methyl acetate	0.02	0.01	0.02	0.01	0.09	0.02	0.07	0.02	0.01	0.00	0.01	0.09	0.02	0.00	0.02	0.00
Ethyl acetate	0.65	0.07	0.69	0.12	0.99	0.18	3.58	2.46	0.28	0.09	0.29	1.61	0.50	0.02	0.44	0.19
3-Methylbutyl acetate	0.02	0.00	0.02	0.00	0.02	0.00	0.01	0.00	0.04	0.01	0.04	0.02	0.04	0.00	0.03	0.01
Ethyl 2-hydroxypropanoate	0.47	0.41	0.20	0.05	0.27	0.09	0.23	0.10	0.11	0.02	0.04	0.13	0.11	0.04	0.09	0.02
Ethyl decanoate	0.05	0.03	0.03	0.00	0.04	0.01	0.03	0.00	0.03	0.00	0.04	0.02	0.03	0.02	0.02	0.00
Sum of Esters	1.20	0.42	0.96	0.13	1.40	0.20	3.93	2.46	0.46	0.09	0.41	1.62	0.71	0.05	0.60	0.19
Acetic acid	0.46	0.05	0.62	0.18	0.73	0.20	0.73	0.20	1.09	0.32	0.47	0.30	0.85	0.43	0.72	0.20
Propanoic acid	0.03	0.00	0.04	0.01	0.04	0.00	0.04	0.01	0.04	0.00	0.04	0.00	0.05	0.00	0.06	0.01
Butanoic acid	0.22	0.04	0.26	0.03	0.30	0.12	0.29	0.10	0.40	0.02	0.32	0.07	0.37	0.03	0.45	0.08
3-Methylbutanoic acid	0.01	0.00	0.13	0.04	0.07	0.04	0.10	0.03	0.02	0.00	0.02	0.01	0.03	0.01	0.08	0.02
Hexanoic acid	0.08	0.01	0.19	0.05	0.06	0.01	0.12	0.05	0.28	0.07	0.18	0.04	0.24	0.06	0.18	0.03
Octanoic acid	0.03	0.00	0.04	0.04	0.02	0.01	0.03	0.02	0.19	0.03	0.14	0.04	0.08	0.01	0.09	0.02
Nonanoic acid	0.06	0.00	0.07	0.09	0.05	0.01	0.06	0.03	0.37	0.08	0.26	0.08	0.15	0.01	0.17	0.03
Sum of Acids	0.90	0.07	1.37	0.22	1.28	0.24	1.37	0.23	2.39	0.34	1.43	0.33	1.77	0.44	1.76	0.22
a-Pinene	0.27	0.08	0.65	0.24	0.22	0.06	0.12	0.02	0.19	0.05	0.22	0.13	0.86	0.26	0.47	0.11
b-Pinene	0.03	0.00	0.06	0.02	0.03	0.01	0.02	0.00	0.03	0.01	0.04	0.01	0.09	0.01	0.06	0.01
Thuja-2,4(10)-diene	0.02	0.00	0.02	0.00	0.02	0.00	0.01	0.00	0.04	0.00	0.04	0.02	0.04	0.00	0.03	0.00
3-Carene	0.04	0.00	0.05	0.01	0.03	0.01	0.03	0.00	0.06	0.01	0.07	0.01	0.10	0.01	0.07	0.01
b-Myrcene	0.05	0.00	0.06	0.01	0.04	0.01	0.04	0.01	0.09	0.02	0.01	0.01	0.13	0.02	0.09	0.01
D-Limonene	1.82	0.20	1.64	0.09	1.53	0.19	1.63	0.41	3.30	0.42	3.59	1.55	3.43	0.20	2.61	0.39

Compound	January								July							
	Day 3		Day 5		Day 7		Day 9		Day 3		Day 5		Day 7		Day 9	
g-Terpinene	0.08	0.03	0.17	0.07	0.06	0.02	0.03	0.00	0.03	0.02	0.05	0.00	0.14	0.05	0.10	0.02
p-Cymene	0.27	0.07	0.50	0.21	0.18	0.05	0.10	0.01	0.14	0.06	0.20	0.08	0.62	0.25	0.39	0.10
Terpene_alcohol_1476	0.08	0.02	0.24	0.10	0.07	0.02	0.05	0.00	0.07	0.01	0.06	0.02	0.09	0.04	0.05	0.00
b-Gurjunene	0.15	0.01	0.21	0.05	0.14	0.02	0.10	0.01	0.19	0.05	0.27	0.04	0.36	0.06	0.31	0.06
Calamenene	0.04	0.00	0.05	0.00	0.04	0.00	0.03	0.01	0.07	0.01	0.09	0.01	0.09	0.02	0.08	0.01
Sum of Terpenoids	2.85	0.23	3.66	0.36	2.36	0.21	2.16	0.41	4.21	0.43	4.62	1.56	5.96	0.42	4.26	0.42
Trimethylamine	1.62	2.62	0.20	0.21	2.46	1.84	5.39	0.52	0.30	0.18	2.38	1.98	0.40	0.32	6.84	1.59
2-Ethylfuran	0.08	0.02	0.07	0.01	0.06	0.01	0.07	0.01	0.09	0.01	0.07	0.02	0.08	0.01	0.09	0.01
2-Pentylfuran	0.02	0.00	0.03	0.01	0.02	0.00	0.01	0.00	0.04	0.02	0.06	0.04	0.05	0.00	0.03	0.02
2,4,6-Trimethylpyridine	0.14	0.02	0.09	0.03	0.15	0.12	0.06	0.04	0.06	0.01	0.07	0.00	0.15	0.10	0.07	0.01
Sum of Miscellaneous	1.86	2.62	0.39	0.21	2.68	1.85	5.53	0.52	0.48	0.18	2.57	1.98	0.69	0.33	7.04	1.59

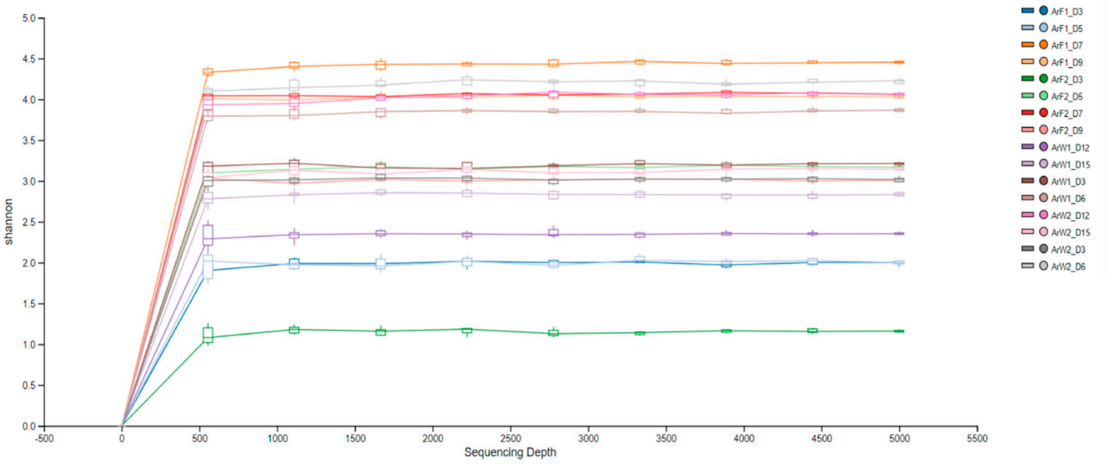

Figure S1. Shannon-Wiener rarefaction curves of whole (ArW) and filleted (ArF) chill-stored meagre caught in January (1) and July (2) at intervals of storage time, obtained through 10 sampling depths.

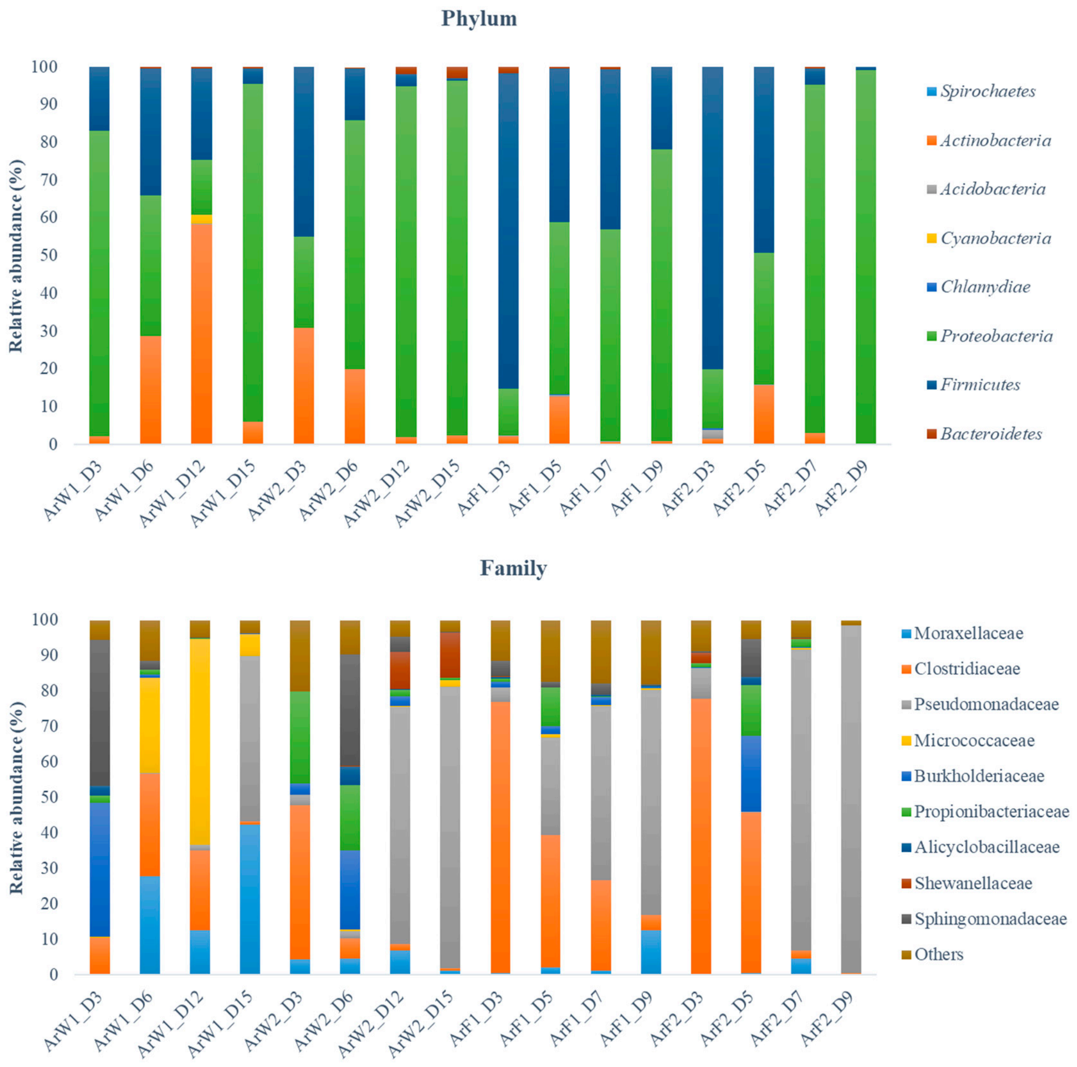

Figure S2. Relative abundance (%) of bacterial phyla (upper) and families (down) of whole (ArW) and filleted (ArF) chill-stored meagre caught in January (1) and July (2), obtained through metabarcoding analysis of 16S rRNA gene at intervals of storage time.

A

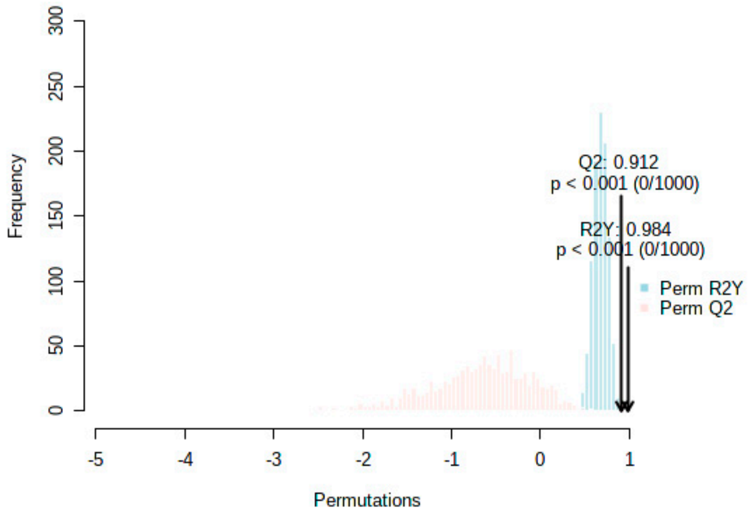

B

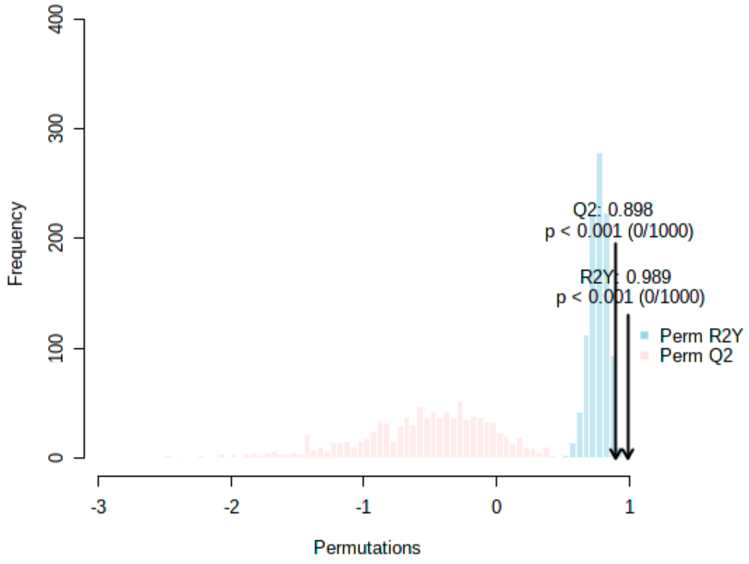

Figure S3. Permutation analysis showing the observed and cross-validated R2Y and Q2 coefficients of OPLS-DA model for (A) whole and (B) filleted meagre.

A

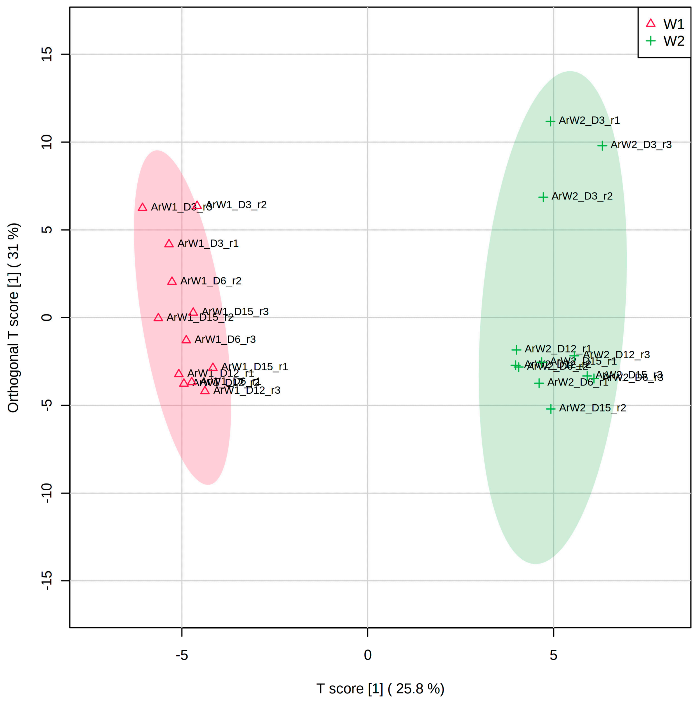

B

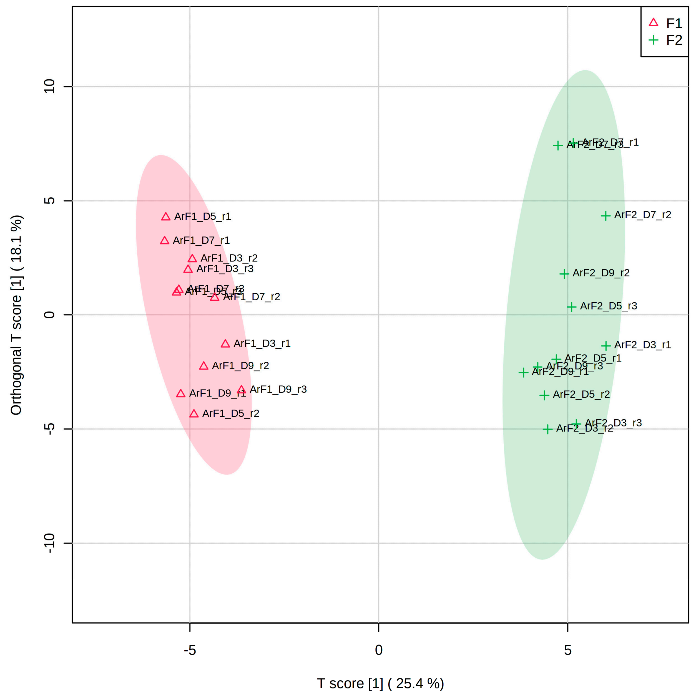

Figure S4. Effect of seasonal variation on the volatile compounds of meagre. OPLS-DA score plot of (A) whole meagre caught in January (ArW1) and July (ArW2), (B) filleted meagre caught in January (ArF1) and July (ArF2). The percentage of the explained response variance is indicated in parentheses. The shaded ellipses correspond to the 95% confidence regions of each class.
